# Advancing Dimensional Models of Psychopathology in Cancer: Insights From Applying the Hierarchical Taxonomy of Psychopathology (HiTOP)

**DOI:** 10.1002/pon.70533

**Published:** 2026-06-26

**Authors:** Darren Haywood, Jai Carmichael, Miriam K. Forbes, Roman Kotov, Robert F. Krueger, Aidan G. C. Wright, Susan L. Rossell, David Castle, Wendy Lam, Maryam Lustberg, Colin E. Vize, Anna Ugalde, Phyllis Butow, Nicolas H. Hart

**Affiliations:** ^1^ Human Performance Research Centre Faculty of Health INSIGHT Research Institute University of Technology Sydney (UTS) Sydney New South Wales Australia; ^2^ Department of Mental Health St. Vincent's Hospital Melbourne Fitzroy Victoria Australia; ^3^ Department of Psychiatry Melbourne Medical School, Dentistry and Health Sciences University of Melbourne Parkville Victoria Australia; ^4^ School of Population Health Faculty of Health Sciences Curtin University Bentley Western Australia Australia; ^5^ Centre for Mental Health and Brain Sciences Swinburne University of Technology Hawthorn Victoria Australia; ^6^ School of Psychological Sciences Monash University Clayton Victoria Australia; ^7^ School of Psychological Sciences Macquarie University Sydney New South Wales Australia; ^8^ Department of Psychiatry & Behavioral Health Stony Brook University Stony Brook New York USA; ^9^ Department of Psychology University of Minnesota Minneapolis Minnesota USA; ^10^ Department of Psychology University of Michigan Ann Arbor Michigan USA; ^11^ Eisenberg Family Depression Center University of Michigan Ann Arbor Michigan USA; ^12^ InsideOut Institute University of Sydney and Sydney Local Health District Sydney New South Wales Australia; ^13^ Department of Health Tasmanian Centre for Mental Health Service Innovation Hobart Tasmania Australia; ^14^ Department of Psychiatry University of Tasmania Hobart Tasmania Australia; ^15^ Centre for Psycho‐Oncology Research and Training Division of Behavioural Sciences School of Public Health LKS Faculty of Medicine The University of Hong Kong Hong Kong SAR China; ^16^ LKS Faculty of Medicine Jockey Club Institute of Cancer Care The University of Hong Kong Hong Kong SAR China; ^17^ School of Medicine Yale University New Haven Connecticut USA; ^18^ Department of Psychology University of Pittsburgh Pittsburgh Pennsylvania USA; ^19^ School of Nursing and Midwifery Faculty of Health Institute for Health Transformation, Centre for Quality and Patient Safety Research Deakin University Deakin Victoria Australia; ^20^ Psycho‐Oncology Co‐operative Research Group School of Psychology University of Sydney Sydney New South Wales Australia; ^21^ Caring Futures Institute College of Nursing and Health Sciences Flinders University Adelaide South Australia Australia; ^22^ School of Medical and Health Sciences Exercise Medicine Research Institute Edith Cowan University Perth Western Australia Australia; ^23^ Cancer and Palliative Care Outcomes Centre Faculty of Health Queensland University of Technology (QUT) Brisbane Queensland Australia; ^24^ Institute for Health Research University of Notre Dame Australia Perth Western Australia Australia

**Keywords:** assessment, cancer, cancer survivor, DSM, HiTOP, mental health, mental illness, psycho‐oncology, psychopathology, survivorship

## Abstract

**Objective:**

Cancer survivors, defined as those living with or beyond a cancer diagnosis, experience more than double the prevalence of psychopathology when compared to the general population. Categorical diagnostic systems, such as the Diagnostic and Statistical Manual of Mental Disorders (DSM), remain dominant in psycho‐oncology despite concerns about reliability, validity, and clinical utility for this population. Dimensional frameworks, such as the Hierarchical Taxonomy of Psychopathology (HiTOP), offer a more precise alternative; however, they have not yet been widely applied in cancer survivors. Accordingly, the objective of this research was to examine the applicability of HiTOP to cancer survivors.

**Methods:**

Data from 1389 participants in 28 countries (*n* = 728 cancer survivors; *n* = 661 community/psychiatric) were collected using the 405‐item HiTOP‐SR, alongside demographic, clinical, and cancer‐specific measures. The HiTOP‐SR normative sample (*n* = 780) was also used. Analyses included parametric, non‐parametric, and factor analytic approaches.

**Results:**

All HiTOP‐SR scales demonstrated strong homogeneity and reliability in cancer survivors. Cancer survivors showed significant elevations across Internalising and Somatoform spectra, with current cancer associated with additional elevations in domains of Thought Disorder and components of Disinhibited and Antagonistic psychopathology. An 11‐factor model was developed and supported for both cancer and community/psychiatric samples, though the magnitudes of the factor loadings sometimes varied between samples. External validity was strong with theoretically aligned associations.

**Conclusion:**

The HiTOP‐SR appears reliable within cancer survivors and provides utility in quantifying a broad array of psychopathology experienced. The results highlight the potential applicability and utility of HiTOP to improve cancer research and clinical practice in psycho‐oncology.

## 1 Introduction

Every year, more than 20 million people are diagnosed with cancer, with projections from high‐income countries indicating that one in two people will be diagnosed with cancer during their lifetime [1]. Among cancer survivors—defined as those living with or beyond a cancer diagnosis—approximately 40% experience a diagnosable mental illness within any given 12‐month period [2, 3, 4]. Psychopathology in cancer survivors is associated with lower income, job loss, physical comorbidities, and earlier mortality—with these relationships likely reflecting complex bidirectional and multifactorial pathways [5, 6, 7]. These factors, in turn, contribute to cancer's projected global economic impact of > $25 trillion USD from 2020 to 2050 [8]. Accordingly, cancer survivors experiencing psychopathology are recognised as a priority population in numerous national and continental cancer plans, with high‐quality psycho‐oncology care identified as an essential component of person‐centred service provision [9, 10, 11]. Thus, it is imperative that mental health clinicians and researchers worldwide are equipped to deliver high‐quality care and to engage in research that recognises and responds to the unique challenges and experiences associated with cancer [1].

The approach taken to conceptualise psychopathology (i.e., to understand and organise psychological and behavioural pathology) in cancer survivors determines the psycho‐oncology assessment approach, case‐formulation, and treatment planning and provision [12]. There are two approaches commonly used [7]. The first is the traditional categorical‐diagnostic approach, exemplified by structured frameworks such as the Diagnostic and Statistical Manual of Mental Disorders (DSM) and International Classification of Diseases (ICD). The second is a pragmatic symptom‐focused approach, in which clinicians rely on questionnaires and unstructured or semi‐structured clinical interviews to assess psychological distress and symptom domains without necessarily assigning formal psychiatric diagnoses. Although distinct in overarching approach, these approaches do overlap. For example, many questionnaires and clinical interview guides used within the pragmatic symptom‐focused approach are informed by DSM/ICD‐based disorders, assessment using DSM/ICD aligned tools does not necessarily need to result in categorical diagnosis(es), and many questionnaires often used within the pragmatic symptom‐focused approach may also be used to inform categorical diagnoses. Regardless, there are significant weakness with both approaches when used in psycho‐oncology. There are significant validity issues using the traditional categorical‐diagnostic approach for cancer survivors [6, 7, 13], as many symptoms considered diagnostic of mental illness are also common side effects of cancers and their treatments (e.g., fatigue, cognitive slowing, sleep issues and weight changes) [6, 7], leading to potential misdiagnosis [6, 7, 14]. Diagnostic categories also obscure essential information, such as individual‐level symptom profiles, for person‐centred and tailored care [15]. In line with this, only around half of practising mental health clinicians routinely use diagnostic systems, and two in three of those do so only for administrative purposes [15, 16, 17, 18]. Instead, clinicians often use the pragmatic symptom‐focused approach [15, 19, 20], but this approach also has widely recognised and significant weaknesses [6, 7, 20]. In particular, tools used in the pragmatic approach (e.g., Distress Thermometer, Hospital Depression and Anxiety Scale, etc.) lack the breadth and depth to understand the array of mental health challenges cancer survivors may experience [20, 21], and lack an evidence‐based framework to guide data‐informed formulations [6, 7]. In recent years, there has been significant movement toward dimensional approaches of psychopathology measurement and conceptualisation across populations with the goal of developing a structured, comprehensive, and empirically derived framework, in line with the scientific literature, for understanding psychopathology [22, 23]. These frameworks also retain the dimensional assessment of symptoms that is often emphasised in symptom‐focused clinical practice. The most notable of these frameworks has been the Hierarchical Taxonomy of Psychopathology (HiTOP) [22].

In response to the limitations of existing approaches to mental illness assessment and conceptualisation, HiTOP was developed from decades of empirical evidence [22, 24]. HiTOP was empirically‐driven rather than consensus‐developed, as per the available diagnostic systems [22, 24]. In line with scientific data, HiTOP organises psychopathology as higher‐ and lower‐level domains measured on dimensions of severity, and shares the strengths of both the diagnostic approach (i.e., structured and consistent) and the pragmatic approach (i.e., non‐categorical) without their weaknesses [7, 20, 25, 26]. HiTOP enables the assessment of breadth and depth, allowing clinicians to tailor the scope of assessment while having a consistent structure to guide their case formulations and treatment planning. Several purpose‐built HiTOP assessment tools have been developed (such as the HiTOP‐Self Report; HiTOP‐SR) which facilitate HiTOP use in research and clinical practice [27]. HiTOP has been embedded in clinical practice through field trials among clinical services providing general mental healthcare [26], with experimental research supporting its potential to improve clinical practice across case‐conceptualisation, treatment planning, communication, and ease of application in this population [6, 7]. A limited body of literature has begun exploring integrative dimensional frameworks, including limited aspects of the HiTOP framework (i.e., the super‐spectra), to capture variability in psychopathology among cancer survivors, with the aim of examining specific outcomes (i.e., treatment adherence, vaccine hesitancy) [28, 29]—showing promise for these approaches in assisting in the understanding of potential drivers of key behaviours within cancer survivorship. However, HiTOP has never been comprehensively and systematically used and assessed in psycho‐oncology clinical practice and research to support people affected by cancer. Such work is important as scales intended to measure specific HiTOP constructs may not demonstrate the same patterns of covariation observed in general population samples—potentially impacting the observed model structure. This is because cancer‐related effects (e.g., somatic and cognitive concerns) may complicate the measurement and conceptualisation of psychopathology constructs, as they may arise directly from cancer or its treatment rather than be characteristic of psychopathology [6, 7], and cancer‐related experiences (e.g., frequent invasive procedures such as blood draws, injections, and intravenous treatments) may impact the saliency and co‐variance of particular symptoms (i.e., injection phobia). Therefore, to understand the degree to which the principles and learnings from HiTOP can be applied to cancer survivor populations empirical evaluation is required to determine whether the HiTOP model and its associated assessment tools generalise appropriately to this population.

Recently, we have detailed a pathway to facilitate the integration of HiTOP into psycho‐oncology [6, 7]. This pathway has been cited in multiple new national oncology guidelines in Australia as a key area of future clinical implementation and research, demonstrating the field's strong interest in the approach [30, 31]. The initial steps of this pathway will determine the applicability of HiTOP to cancer survivor populations. The assessment of the applicability of HiTOP to cancer survivors requires testing of the reliability and validity of the recently developed HiTOP measure (the HiTOP‐SR [32]) in this population, and of the utility of the measure to detect elevations in psychopathology among cancer survivors, to inform a dimensional model of psychopathology that can apply in cancer survivor and general and psychiatric populations [6, 7].

### 1.1 Aims

This research aims to (1) explore the homogeneity and reliability of the HiTOP‐SR in cancer survivors; (2) examine whether the HiTOP‐SR scales detect elevations in psychopathology among cancer survivors; (3) develop a dimensional model of psychopathology in a combined sample of cancer survivors and community/psychiatric groups; (4) examine whether the same dimensional model of psychopathology works for cancer survivor and community/psychiatric samples (i.e., testing measurement invariance); and (5) explore the external validity of the dimensional model of psychopathology in cancer survivors.

## 2 Methods

### 2.1 Samples

#### 2.1.1 Cancer Survivors

Cancer survivors were recruited to the study via the participant sourcing platform Prolific [33], which is recognised as a valid and reliable platform generating data of comparable quality to that collected in laboratory settings [34, 35, 36, 37] and has been used in thousands of studies worldwide in both psychopathology and cancer (e.g., [38, 39]); it was also the data collection approach taken for the original development, validation, and norm development of the HiTOP‐SR [32].

Cancer survivor participants were required to be 18 years or older, have a current or previous cancer diagnosis of any type or stage, and be fluent in English (reading and writing). The only exclusion criterion was having a neurological condition (except for brain cancer). The study was visible on Prolific only to people 18 years or older who had a cancer diagnosis listed in their Prolific profile, and open to all members from all countries. Participants received compensation based on Prolific's recommended rates in line with survey completion time. Participants were compensated £5.50 for their time, and the median total participation time was 37 minutes. Data collection was approved by the University of Technology Sydney Human Research Ethics Committee (ETH24‐9420).

#### 2.1.2 Community/Psychiatric Sample

To develop a dimensional model of psychopathology across combined cancer and community/psychiatric samples, and to examine measurement non‐invariance of this model, individual participant data were drawn from the baseline survey of the Intensive Longitudinal Investigation of Alternative Diagnostic Dimensions (ILIADD) study [40]. Participants were recruited through a research registry supported by the USA National Institutes of Health's Clinical and Translational Science Institute. Eligibility criteria included being aged 18–50 years and owning a compatible Android or iPhone smartphone. Recruitment into the ILIADD study initially targeted individuals with a recent history of mental health treatment, but was later expanded to include participants regardless of treatment history to ensure greater variability in symptom presentation. Given that this is a mixed community and psychiatric sample, some participants may have been cancer survivors, but likely were mostly not, given the typical prevalence of cancer across populations (e.g., ∼53.5 million people globally, < 1%, of the global population, are estimated to be living within 5 years of diagnosis worldwide [41]).

#### 2.1.3 Normative Sample

To assess the ability of the HiTOP‐SR to detect elevations in psychopathology in cancer survivors, we also used the normative data for the current 405‐item HiTOP‐SR. The normative sample was individuals aged 18–80 years who were recruited through Prolific. Apart from fluency in the English language, there were no other inclusion or exclusion criteria. Procedures for the collection of this data largely matched those of the cancer survivor sample. See Table 1 and https://www.3plab.org/hitop for further details of the normative sample.

**TABLE 1:** Demographic characteristics.

Sample	Cancer survivor sample (*n* = 728)	Community/psychiatric sample (*n* = 661)	Combined cancer and community/psychiatric sample (*n* = 1389)	Normative sample (*n* = 780)
Characteristic	*n* (%)/*M* (SD)			
Age (years)	49.6 (15.2)	30.8 (8.9)	40.7 (15.7)	44.4 (14.9)
Sex				
Female	483 (66.3%)	547 (82.8%)	1030 (74.2%)	394 (50.5%)
Male	244 (33.5%)	113 (17.1%)	357 (25.7%)	379 (48.6%)
Intersex	1 (0.1%)	1 (0.2%)	2 (0.1%)	—
Prefer not to say	—	—	—	7 (0.9%)
Gender identity				
Woman	479 (65.8%)	495 (74.9%)	974 (70.1%)	376 (48.5%)
Man	243 (33.4%)	106 (16.0%)	349 (25.1%)	375 (48.1%)
Non‐binary	5 (0.7%)	24 (3.6%)	29 (2.1%)	11 (1.4%)
Other/multiple	1 (0.1%)	36 (5.4%)	37 (2.7%)	11 (1.4%)
Prefer not to say	—	—	—	5 (0.6%)
Country				
United States	320 (44.0%)	661 (100%)	981 (70.6%)	—
United Kingdom	225 (30.9%)	0 (0.0%)	225 (16.2%)	—
South Africa	49 (6.7%)	0 (0.0%)	49 (3.5%)	—
Other	134 (18.4%)	0 (0.0%)	134 (9.6%)	—
Ethnicity/race				
Caucasian/white	545 (74.9%)	496 (75.0%)	1041 (74.9%)	546 (70.0%)
African/African American	100 (13.7%)	36 (5.4%)	136 (9.8%)	83 (10.6%)
Asian	30 (4.1%)	43 (6.5%)	73 (5.3%)	45 (5.8%)
Multiple	14 (1.9%)	60 (9.1%)	74 (5.3%)	60 (7.7%)
Other	38 (5.2%)	26 (3.9%)	64 (4.6%)	41 (5.3%)
Missing	1 (0.1%)	0 (0.0%)	1 (0.1%)	5 (0.5%)
Primary language				
English	641 (88.0%)	639 (96.7%)	1280 (92.2%)	—
Other	87 (12.0%)	22 (3.3%)	109 (7.8%)	—
Education				
Post‐secondary	593 (81.5%)	612 (92.6%)	1205 (86.8%)	660 (84.6%)
Secondary school	133 (18.3%)	49 (7.4%)	182 (13.1%)	112 (14.4%)
Primary school or less	2 (0.3%)	0 (0.0%)	2 (0.1%)	6 (0.8%)
Prefer not to say	—	—	—	2 (0.3%)
Employment				
Employed full‐time	345 (47.4%)	332 (50.2%)	677 (48.7%)	379 (48.6%)
Employed part‐time	142 (19.5%)	138 (20.9%)	280 (20.2%)	149 (19.1%)
Unemployed	64 (8.8%)	67 (10.1%)	131 (9.4%)	148 (18.9%)
Student	23 (3.2%)	36 (5.4%)	59 (4.2%)	—
Retired	113 (15.5%)	0 (0.0%)	113 (8.1%)	83 (10.6%)
Home duties	24 (3.3%)	16 (2.4%)	40 (2.9%)	—
Other	17 (2.3%)	72 (10.9%)	89 (6.4%)	—
Prefer not to say	—	—	—	21 (2.7%)
Mental health treatment history				
Currently receiving treatment	—	415 (62.7%)	—	158 (20.3%)
Ever received treatment	—	530 (80.2%)	—	492 (63.5%)
Received treatment in the previous 2 years, but not currently	—	106 (20.0%)	—	124 (15.9%)

*Note:* Categories reflect the harmonisation of demographic data independently collected across the three samples.

### 2.2 Measures

#### 2.2.1 Demographic and Clinical History Information

Participants completed brief self‐report measures of demographic characteristics and clinical history. Cancer survivors reported key demographic information and cancer‐related history, including cancer type, stage, and treatment. Participants in the ILIADD study and HiTOP‐SR normative sample completed baseline measures assessing core demographics and prior mental health treatment.

#### 2.2.2 HiTOP‐SR

Dimensional psychopathology was assessed in all samples using development versions of the HiTOP‐SR inventory. The HiTOP‐SR [27, 32] was developed by the HiTOP Consortium's Measures Development Workgroup to capture a wide range of psychopathology symptoms and behaviours represented within the HiTOP model. Cancer survivors and the normative sample completed the current publicly available version of the HiTOP‐SR (https://www.3plab.org/hitop), which comprised 405 items organised into 87 unique scales. This version excluded the harmful substance use module, which remains under development. The HiTOP‐SR asks respondents to rate how much each item has applied to them over the past 12 months, using a Likert scale ranging from 1 (‘Not at all’) to 4 (‘A lot’). The scales have been provisionally organised under the HiTOP spectra of detachment, externalising, internalising, somatoform, and thought disorder based on the existing HiTOP structure. Ongoing psychometric studies continue to evaluate the internal structure of the HiTOP‐SR [42, 43, 44].

The ILIADD study employed an earlier, unreleased version of the HiTOP‐SR. Consequently, 21 items administered to the cancer survivor sample were not included in the ILIADD study–this included all items from the cleaning and hallucinations scales, and two of four items from the premature orgasm scale (see Supporting Information S1: Table S1 for details). These three scales were therefore excluded from analyses involving the combined cancer survivor and community/psychiatric samples, which used a harmonised set of 384 items spanning 83 unique scales administered across both datasets.

#### 2.2.3 Fear of Cancer Recurrence and Progression

Given their status as among the most widely studied and clinically significant psychological concerns in psycho‐oncology [45], fear of cancer recurrence and fear of progression were included as external validation measures. These constructs were selected to examine how established cancer‐specific psychological experiences relate to broader dimensions of psychopathology within the HiTOP framework. To this end, cancer survivors also completed either the Fear of Progression‐Questionnaire Rapid Screener (FoP‐Q‐RS) [46] or the Fear of Cancer Recurrence Screening Measure (FCR‐1r) [47]. Participants who reported currently having cancer completed the FoP‐Q‐RS, and those who reported previously having cancer completed the FCR‐1r. The FoP‐Q‐RS screens for fear of cancer progression severity [46] and consists of five items, each rated on a five‐point Likert scale ranging from 1 (‘never’) to 5 (‘very often’), with total scores ranging from 5 to 25. Higher scores indicate greater fear of cancer progression. The FoP‐Q‐RS showed good‐to‐excellent internal consistency in our cancer survivor sample (*α* = 0.84). The FCR‐1r is a single‐item screening tool to assess the severity of fear of cancer recurrence among cancer survivors [47]. Respondents rate their level of fear on a 0–100 visual analogue scale (0 = no fear of recurrence, 100 = worst possible fear of recurrence), where higher scores indicate greater fear.

#### 2.2.4 Physical Functioning

Cancer survivors completed the Physical Functioning subscale of the Short Form‐36 Health Survey [48]. The 10‐item Physical Functioning subscale is a collection of self‐report items designed to assess limitations in physical functioning due to health problems. Responses were scored on a 3‐point scale indicating whether the individual is limited a lot, a little, or not at all. Raw scores were summed, providing a 10–30 scale, with higher scores reflecting better physical functioning. The Physical Functioning subscale showed excellent internal consistency in our cancer survivor sample (*α* = 0.93).

### 2.3 Data Analysis

Analyses were conducted in the R statistical software environment (version 4.3.2), including the packages *psych* (version 2.4.12) and *lavaan* (version 0.6–19) [49, 50]. Frequencies and descriptive analyses were used to describe demographic and clinical information of the samples.

#### 2.3.1 Aim 1: Homogeneity and Reliability of HiTOP‐SR Scales in Cancer Survivors

Homogeneity and reliability of the HiTOP‐SR scales in cancer survivors were evaluated using all 87 HiTOP‐SR scales plus slightly modified versions of the scales that were subsequently used for the joint modelling of psychopathology in the combined cancer survivor and community/psychiatric sample (as described earlier; Supporting Information S1: Table S1). This aim was addressed within a confirmatory factor analysis (CFA) framework. In the cancer survivor sample, each HiTOP‐SR scale was evaluated using a one‐factor ordinal CFA model estimated with the weighted least squares mean and variance adjusted (WLSMV) method, based on the polychoric correlation matrix. To assess scale homogeneity, we used the unbiased standardised root mean squared residual (uSRMR) and the absolute largest residual correlation between items [44, 51, 52]. A one‐factor model delivered adequate scale homogeneity as the uSRMR was not significantly greater than 0.064, and the largest residual correlation was not significantly greater than 0.15, using 90% confidence intervals (i.e., one‐sided statistical test with a 5% probability of error) [44]. Reliability of each scale was assessed using McDonald's omega (*ω*) coefficient, with values exceeding 0.75 considered acceptable [53, 54, 55, 56]. Note that, for the antisocial behaviour scale, we collapsed item responses 3 (‘Moderately’) and 4 (‘A lot’) due to sparse cells and model estimation issues.

#### 2.3.2 Aim 2: Elevations in Psychopathology Among Cancer Survivors

To examine whether the HiTOP‐SR scales detected elevations in psychopathology among cancer survivors, each scale in the cancer survivor sample was compared to the HiTOP‐SR normative data. Comparisons with the available community/psychiatric sample were not performed, as that sample included a non‐normative proportion of treatment‐seeking individuals. Each scale was analysed separately using two‐sided Welch's independent‐samples *t*‐tests, which are robust to violations of homogeneity of variance, with an alpha level of 0.05. To reduce the risk of false positives due to multiple testing, Benjamini‐Hochberg corrections were applied separately within each set of ‘family’ of normative comparisons (overall cancer survivor sample, current cancer sample, and past cancer sample). Cohen's *d* values were calculated to quantify the magnitude of observed differences. Pairwise comparisons were conducted among three groups: (1) participants in the cancer survivor sample with current cancer, (2) participants in the cancer survivor sample with past cancer, and (3) the normative sample.

#### 2.3.3 Aim 3: Dimensional Model in Cancer Survivor and Community/Psychiatric Groups

A dimensional model of psychopathology was constructed using the cancer survivor and community/psychiatric sample combined. Minor modifications were applied to include only the 83 scales and their corresponding items available in both samples (Supporting Information S1: Table S1). An exploratory factor analysis (EFA) was conducted on a Spearman's correlation matrix to accommodate positive skew in some scales. An oblique (Oblimin) rotation was applied, reflecting the expectation that the extracted factors would be intercorrelated. The number of factors to extract was determined using both parallel analysis and Velicer's minimum average partial (MAP) criterion [57, 58]. The most comprehensive, fine‐grained solution was selected—within the limits supported by these methods—in which all factors were well‐defined. Following the conventions set in previous studies [59, 60, 61, 62], a factor was considered well‐defined if it had at least three primary indicators, with a primary loading of ≥|0.30| and no cross‐loading within 0.10 of that primary loading.

#### 2.3.4 Aim 4: Measurement Invariance Across Groups

To ascertain whether the psychopathology dimensions identified above were invariant between the cancer survivor and non‐cancer comparison samples, configural invariance and metric invariance were the focus, due to their clinical relevance. Configural invariance assesses whether the underlying factor structure is consistent across groups, whereas metric invariance examines whether the relationship of the specific indicators (i.e., HiTOP‐SR scales) to their corresponding latent factor differs between groups. Both forms of invariance were evaluated using multi‐group CFAs with robust maximum likelihood (MLR) estimation. Due to convergence issues when fitting the full dimensional model simultaneously, CFAs were estimated for one dimension at a time; indicators with loadings ≥ |0.30| from the original EFA were used to specify a one‐factor CFA per psychopathology dimension. Configural invariance was assessed using the same indices described earlier—uSRMR, the largest residual correlation between items, and the omega (*ω*) reliability coefficient. Adequate model fit (uSRMR significantly greater than 0.064 and largest residual correlation not significantly greater than 0.15) and reliability indices (*ω* > 0.75) for both groups were interpreted as supporting configural invariance. While omega is not a direct test of configural invariance, we retained it here because it provides complementary information regarding the extent to which the HiTOP‐SR scales within each group reflect a common underlying factor. Metric invariance was tested by comparing the configural and metric models (with unconstrained vs. constrained factor loadings between groups, respectively) using chi‐square difference tests (*α* = 0.05) [63]. Failure to reject the null hypothesis (*p* > 0.05) indicated support for metric invariance. Additionally, changes in model fit indices were examined: if the root mean square error of approximation (RMSEA) increased by more than 0.015, or the comparative fit index (CFI) decreased by more than 0.01 from the configural to metric model, the additional misfit was considered too large to support metric invariance [57, 58]. In such cases, factor loadings differing by ≥ 0.10 were inspected, which may indicate that corresponding scales differed in loadings between groups.

#### 2.3.5 Aim 5: External Validity of the Dimensional Model in Cancer Survivors

An evaluation of the external validity of the dimensional model of psychopathology among cancer survivors was performed. In this step, associations between the identified psychopathology dimensions and cancer‐specific variables were examined. Psychopathology dimension scores were calculated for participants in the cancer survivor sample as regression‐based factor scores, extracted from the one‐factor CFA models assessing configural invariance (i.e., where factor loadings were not constrained to be equal across groups), thereby drawing on factor loadings specific to the cancer group. Factor determinacy (i.e., the correlation between factor score estimates and true latent factors) ranged from 0.83 to 0.97. Values ≥ 0.80 have been deemed adequate for research purposes, without substantial indeterminacy that would risk inconsistent ordering of participants along the latent dimension [64, 65], and all but two factors surpassed the stricter 0.90 threshold recommended for adequate individual‐level validity [64, 66, 67]. First, factor scores along the psychopathology dimensions between participants with a current versus past cancer diagnosis were compared (Welch's *t*‐tests and Cohen's *d*). Next, within the full cancer survivor sample and each of these subgroups, associations between psychopathology dimensions and other clinically important variables were examined: physical functioning, fear of cancer progression (for those with current cancer only), fear of cancer recurrence (for those with past cancer only), and time since cancer diagnosis. To control for multiple testing, we applied Benjamini‐Hochberg corrections separately for each family of analyses (i.e., each external variable).

## 3 Results

### 3.1 Demographic and Clinical Characteristics

Demographic characteristics of cancer survivor, community/psychiatric, and normative samples are presented in Table 1.

Clinical history of the cancer survivor sample is provided in Table 2.

**TABLE 2:** Clinical history of cancer survivor sample (*n* = 728).

Characteristic	*n* (%)	*M* (SD)	Range
Cancer status			
Current has cancer	155 (21.3%)	—	—
Previously had cancer	573 (78.7%)	—	—
Cancer type (primary or secondary)			
Breast	186 (25.5%)	—	—
Lymphoma	72 (9.9%)	—	—
Melanoma	62 (8.5%)	—	—
Prostate	59 (8.1%)	—	—
Leukaemia	58 (8.0%)	—	—
Bowel/colorectal	55 (7.6%)	—	—
Thyroid	49 (6.7%)	—	—
Cervical	43 (5.9%)	—	—
Ovarian	33 (4.5%)	—	—
Lung	30 (4.1%)	—	—
Testicular	30 (4.1%)	—	—
Uterine/endometrial	26 (3.6%)	—	—
Skin (non‐melanoma)	24 (3.4%)	—	—
Other	126 (17.3%)	—	—
Cancer stage—Current			
No current cancer	573 (78.7%)	—	—
Stage I	77 (10.6%)	—	—
Stage II	42 (5.8%)	—	—
Stage III	22 (3.0%)	—	—
Stage IV	14 (1.9%)	—	—
Cancer stage—At most advanced			
Stage I	331 (44.5%)	—	—
Stage II	244 (33.5%)	—	—
Stage III	113 (15.5%)	—	—
Stage IV	40 (5.5%)	—	—
Cancer treatments received—Ever			
Chemotherapy	372 (51.1%)	—	—
Radiation therapy	278 (38.2%)	—	—
Surgery/excision	263 (36.1%)	—	—
Hormone therapy	161 (22.1%)	—	—
Immunotherapy	82 (11.3%)	—	—
Targeted therapy	64 (8.8%)	—	—
Other	26 (3.6%)	—	—
None	25 (3.4%)	—	—
Cancer treatments receiving—Current			
Chemotherapy	73 (10.0%)	—	—
Radiation therapy	32 (4.4%)	—	—
Surgery/excision	5 (0.7%)	—	—
Hormone therapy	105 (14.4%)	—	—
Immunotherapy	29 (4.0%)	—	—
Targeted therapy	39 (5.4%)	—	—
Other	14 (1.9%)	—	—
None	503 (69.1%)	—	—
Years since original cancer diagnosis (*n* = 727)	—	10.2 (10.4)	0.02–72.1
Fear of cancer progression (*n* = 155)	—	15.9 (6.0)	5–25
Fear of cancer recurrence (*n* = 573)	—	51.8 (30.7)	0–100
Physical functioning (*n* = 728)	—	24.6 (5.4)	10–30

*Note:* Percentages for cancer type exceed 100% because they include both primary and secondary cancers. One participant's time since diagnosis was coded as missing because the reported date of diagnosis occurred after the survey completion date. Fear of cancer progression was measured using the Fear of Progression‐Questionnaire Rapid Screener (FoP‐Q‐RS), which yields scores ranging from 5 to 25 (higher scores indicate greater fear of progression). Fear of cancer recurrence was measured using the Fear of Cancer Recurrence Screening Measure (FCR‐1r), yielding scores from 0 to 100 (higher scores indicate greater fear of recurrence). Physical functioning was measured using the Physical Functioning subscale of the Short Form‐36 Health Survey. Raw responses to the relevant items were summed to generate total scores ranging from 10 to 30, with higher scores indicating better physical functioning.

### 3.2 Homogeneity and Reliability of HiTOP‐SR Scales in Cancer Survivors

According to a priori criteria, all HiTOP‐SR scales demonstrated sufficient homogeneity (uSRMR = 0.000–0.067, absolute largest residual correlations < 0.17) and reliability (*ω* = 0.79 to > 0.99), each effectively measuring a specific, homogeneous component of psychopathology in the cancer survivor sample. The statistical output from the CFAs testing homogeneity and reliability of the scales in cancer survivors is located in Supporting Information S1: Table S2.

### 3.3 Elevations in Psychopathology Among Cancer Survivors

**FIGURE 1:**
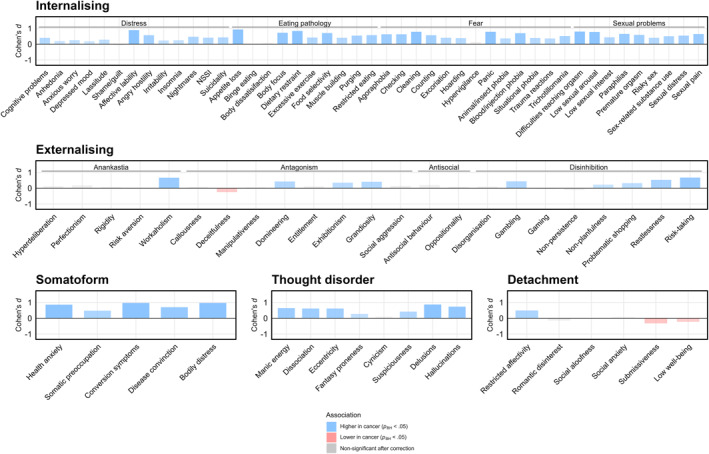
HiTOP‐sr current cancer and normative data comparisons. HiTOP‐SR scale scores in participants with current cancer against the HiTOP‐SR normative data. Scales follow the rational organisation of the HiTOP‐SR in relation to the established HiTOP model.

**FIGURE 2:**
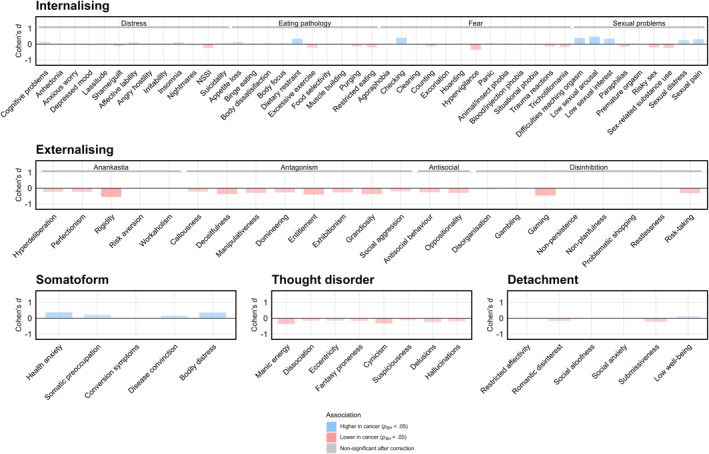
HiTOP‐SR past cancer and normative data comparisons. HiTOP‐SR scale scores in participants with current cancer against the HiTOP‐SR normative data. Scales follow the rational organisation of the HiTOP‐SR in relation to the established HiTOP model.

Comparisons between cancer survivors' scores on each HiTOP‐SR scale and the available normative data are presented in Figures 1 and 2. For Brevity, results for participants with current cancer and past cancer are presented in the main manuscript, while findings for the overall cancer survivor sample are provided in Supporting Information S1: Figure S1. These figures follow the rational organisation of the HiTOP‐SR scales from the HiTOP Consortium's Measures Development Workgroup (since the empirical structure of the HiTOP‐SR is still an area of active investigation), thereby highlighting where within the established HiTOP model cancer survivors may show elevations in psychopathology.

In sum, the overall cancer survivor sample (current cancer and previous cancer) showed elevated scores across the established HiTOP internalising and somatoform spectra, mostly with small standardised effect sizes, alongside small reductions in *Externalising* components and minimal differences in *Thought Disorder* and *Detachment* (Supporting Information S1: Figure S1). However, as shown in Figure 1, those participants with current cancer showed much more pronounced elevations across *Internalising* (small‐to‐large), *Somatoform* (medium‐to‐large), and *Thought Disorder* spectra (mostly medium), alongside several small‐to‐medium elevations in *Disinhibited Externalising* (risk‐taking, restlessness, gambling, problematic‐shopping, and non‐planfulness) and *Antagonistic Externalising* components (domineering, grandiosity, and exhibitionism). In particular, large elevations were seen in the homogeneous components of *Bodily Distress* (*d* = 0.97, *p* < 0.001), *Conversion Symptoms* (*d* = 0.97, *p* < 0.001), *Appetite Loss* (*d* = 0.93, *p* < 0.001), *Affective Lability* (*d* = 0.89, *p* < 0.001), *Delusions* (*d* = 0.87, *p* < 0.001), *Health Anxiety* (*d* = 0.86, *p* < 0.001), and *Dietary Restraint* (*d* = 0.84, *p* < 0.001).

In contrast (Figure 2), those with past cancer endorsed items mapping onto *Externalising* and *Thought Disorder* spectra less frequently than the normative sample (mostly small standardised effect sizes), but still had small elevations in the *Somatoform* spectrum. Results for the *Internalising* spectrum were more mixed in those with past cancer, with some components endorsed significantly more often than norms, and others significantly less, with elevations within this spectrum largely isolated to the established HiTOP subfactor of *Sexual Problems* (small differences).

### 3.4 Dimensional Model in Cancer and Community/Psychiatric Groups

Parallel analysis and Velicer's MAP suggested 14 and 11 underlying dimensions respectively as optimal in accounting for interrelationships among the 83 HiTOP‐SR scales available in the combined cancer survivor and community/psychiatric sample. Therefore, solutions with up to 14 factors were considered, but the most fine‐grained solution in which all factors were well‐defined (i.e., with a coherent pattern of loadings and at least three indicators per factor) contained 11 factors. The 13‐ and 14‐factor solutions each contained poorly defined factors with fewer than three primary indicators. The 12‐factor solution produced an additional factor comprising body dissatisfaction, problematic shopping, and binge eating; however, this factor was difficult to interpret substantively, particularly given that binge eating also loaded with similar magnitude but in the opposite direction on a factor relating to low appetite and intake, where its placement was more theoretically consistent with the broader eating‐related content of the dimension. Consequently, the 11‐factor solution was retained as the most fine‐grained and interpretable representation of the data.

**FIGURE 3:**
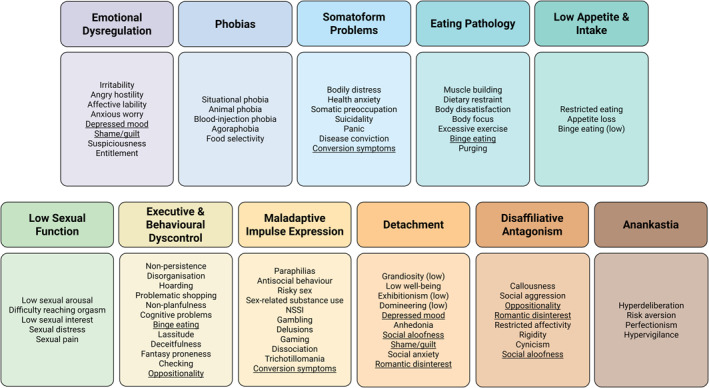
Dimensional model of psychopathology in the combined cancer survivor and community/psychiatric sample. Listed HiTOP‐SR scales had loadings ≥ |0.30| on the corresponding factor and therefore were considered to index that underlying dimension. Scales appear in descending order of factor loading. Underlined scales cross‐loaded in the same direction on multiple factors. Scales marked ‘(low)’ were reverse indicators of (i.e., loaded negatively on) the factor.

Guided by the pattern of factor loadings and the established HiTOP model, these 11 correlated dimensions were labelled *Emotional Dysregulation*, *Phobias*, *Somatoform Problems*, *Eating Pathology*, *Low Appetite & Intake*, *Low Sexual Function*, *Executive & Behavioural Dyscontrol*, *Maladaptive Impulse Expression*, *Detachment*, *Disaffiliative Antagonism*, and *Anankastia*. The 11‐factor dimensional model of psychopathology and HiTOP‐SR scales that index each dimension are presented in Figure 3. The full factor loading matrix is provided in Supporting Information S1: Table S3. Correlations among the 11 factors are reported in Supporting Information S1: Table S4.

### 3.5 Measurement Invariance Across Cancer Survivor and Community/Psychiatric Groups

Multi‐group confirmatory factor models were estimated one dimension at a time, given convergence problems when attempting to fit all 11 dimensions simultaneously. All one‐factor CFA models converged, except for *Low Appetite & Intake*. This dimension had only three indicators and likely failed to converge due to insufficient degrees of freedom. As an alternative, one‐factor EFAs were fit to these three indicators in each sample; the resulting factor loadings were inspected qualitatively, and coefficient alpha (a reliability estimate) was computed for the summed total score.

Regarding configural invariance, the one‐factor CFA models for *Phobias*, *Somatoform Problems*, and *Maladaptive Impulse Expression* fit well in both groups, indicating that the indicators for each dimension clustered together consistently regardless of participants being cancer survivors or from the community/psychiatric sample. For the remaining eight dimensions, one or more indices of fit or reliability (i.e., uSRMR, largest absolute residual correlation, omega reliability) failed to meet our a priori criteria in one or both groups. However, closer inspection suggested that CFA model misfit occurred similarly across groups rather than representing true configural non‐invariance. For example, poor fit of the *Detachment* dimension stemmed from including the three extraverted traits as reverse indicators—a pattern seen in both samples; reliability was similarly poor in both groups for *Low Appetite & Intake* due to the inclusion of binge eating as a reverse indicator. Overall, evidence for configural invariance was strongest for *Phobias*, *Somatoform Problems*, and *Maladaptive Impulse Expression*, which met all a priori criteria in both groups. For the remaining dimensions, interpretation was complicated by one or more homogeneity or reliability indices failing to meet prespecified criteria. However, the pattern of model misfit appeared broadly similar across groups, suggesting that these findings may reflect limitations associated with imposing more restrictive one‐factor CFA models onto a structure derived from the 11‐factor exploratory correlated‐factors model, the latter of which allowed for cross‐loadings. The full configural invariance results are presented in Supporting Information S1: Table S5.

The 11 psychopathology dimensions were then tested for metric invariance. *Executive and Behavioural Dyscontrol* showed metric invariance across all indices (i.e., *χ* ^2^ difference test, ΔCFI, ΔRMSEA), indicating that the HiTOP‐SR scales indexing this dimension had similar loadings in both the cancer survivor and community/psychiatric samples. *Low Sexual Function* also showed no strong evidence of metric non‐invariance, despite a ΔRMSEA > 0.015, with no factor loadings differing by ≥ 0.10 between samples. Similarly, despite a significant *χ* ^2^ difference test, *Emotional Dysregulation* showed minimal ΔCFI and ΔRMSEA, with only the entitlement scale (a relatively weak indicator of this dimension) having a loading differing by ≥ 0.10 between groups.

For other dimensions, there were more notable differences in indicator loadings across groups: blood‐injection phobia loaded more strongly on *Phobias* in the cancer survivor sample (*λ*: cancer = 0.68, community/psychiatric = 0.46); bodily distress was weaker on *Somatoform Problems* (*λ*: cancer = 0.75, community/psychiatric = 0.86); excessive exercise (*λ*: cancer = 0.66, community/psychiatric = 0.53) and purging (*λ*: cancer = 0.59, community/psychiatric = 0.38) were stronger, but dietary restraint weaker (*λ*: cancer = 0.51, community/psychiatric = 0.69), on *Eating Pathology*; grandiosity (*λ*: cancer = −0.19, community/psychiatric = −0.55) and low well‐being (*λ*: cancer = 0.51, community/psychiatric = 0.72) weaker on *Detachment*; callousness (*λ*: cancer = 0.62, community/psychiatric = 0.78) and social aloofness (λ: cancer = 0.47, community/psychiatric = 0.62) weaker on *Disaffiliative Antagonism*; and perfectionism (*λ*: cancer = 0.55, community/psychiatric = 0.40) and hypervigilance (*λ*: cancer = 0.60, community/psychiatric = 0.49) stronger, but risk aversion weaker (*λ*: cancer = 0.62, community/psychiatric = 0.76), on *Anankastia*. Many indicators were also stronger on *Maladaptive Impulse Expression* in cancer survivors (paraphilias, risky sex, sex‐related substance use, NSSI, gambling, delusions, gaming, and trichotillomania), though this may have reflected low endorsement of these indicators, especially in the community/psychiatric sample. The full metric invariance results are presented in Supporting Information S1: Table S6.

### 3.6 External Validity of the Dimensional Model in Cancer Survivors

**FIGURE 4:**
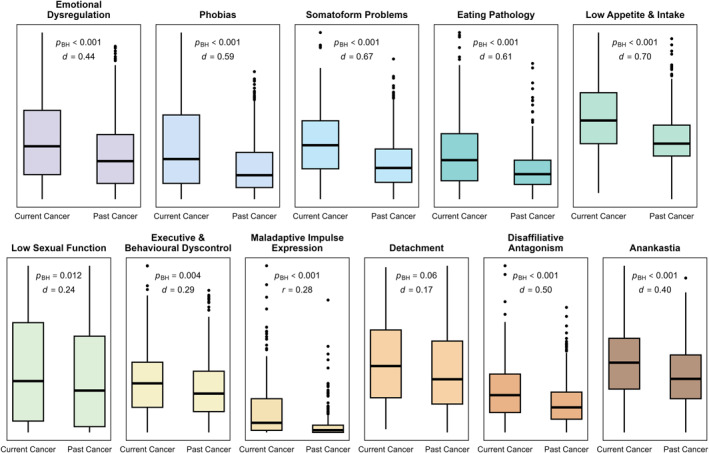
Comparison of psychopathology dimension scores between participants with current versus previous cancer. Current cancer *n* = 155, previous cancer *n* = 573. Psychopathology dimension scores were calculated as CFA‐derived factor score estimates using loadings specific to the cancer survivor sample. The exception was *Low Appetite & Intake* dimension, for which the one‐factor CFA model failed to converge; in this case, the unweighted mean of the three HiTOP‐SR scales indexing the dimension was used as a pragmatic composite score. Group comparisons were conducted using welch's independent‐samples *t*‐tests, except for maladaptive impulse expression, which exhibited substantial skewness (*Z* = 3.83) and kurtosis (*Z* = 17.60); for this dimension, the difference was evaluated using the wilcoxon rank‐sum test with *r* as the effect size.

**FIGURE 5:**
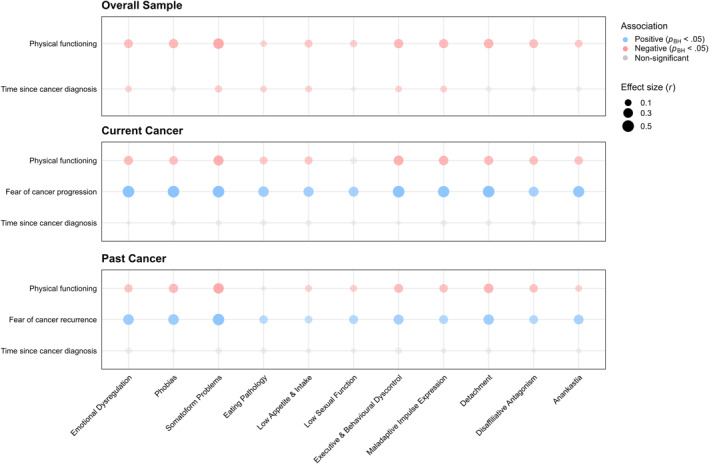
Associations between psychopathology dimensions and cancer‐specific variables. Associations between psychopathology dimensions and cancer‐specific variables among cancer survivors, including the overall cancer survivor sample (top), those with current cancer (middle), and those with past cancer (bottom).

Differences in dimensional psychopathology scores between participants with current versus past cancer are presented in Figure 4, with the associations between psychopathology scores and other cancer‐specific variables, adjusted for multiple testing presented in Figure 5. Associations were examined using factor score estimates, based on loadings specific to the cancer survivor sample, except for the *Low Appetite & Intake* dimension, where the one‐factor CFA model failed to converge. For this dimension, the unweighted average of the three HiTOP‐SR scales indexing the dimension was used as a pragmatic composite score.

Participants with current cancer showed significantly higher scores on all psychopathology dimensions than those with past cancer (*p*_BH_ < 0.001–0.012), with small‐to‐medium standardised effect sizes (ES = 0.24–0.70), except for *Detachment*, for which there was no significant difference (*p*_BH_ = 0.060). The largest differences were seen in *Low Appetite & Intake* (*d* = 0.70), *Somatoform Problems* (*d* = 0.67), *Eating Pathology* (*d* = 0.61), *Phobias* (*d* = 0.59), and *Disaffiliative Antagonism* (*d* = 0.50).

Poorer physical functioning was consistently associated with higher psychopathology in survivors (*p*_BH_ < 0.001–0.046), generally with small effect sizes (ES = −0.08 to −0.38). The *Somatoform Problems* dimension showed the strongest associations across the full cancer sample (*r* = −0.38, *p*_BH_ < 0.001), as well as the subgroups with current (*r* = −0.34, *p*_BH_ < 0.001) or past cancer (*r* = −0.37, *p*_BH_ < 0.001). The only non‐significant associations here were between *Low Sexual Function* and physical functioning in the current cancer subgroup (*p*_BH_ = 0.223), and between *Eating Pathology* and physical functioning in the past cancer subgroup (*p*_BH_ = 0.540).

Greater fear of cancer progression was associated with all psychopathology dimensions in the current cancer subgroup (*p*_BH_ < 0.001), generally with medium‐to‐large effect sizes (ES = 0.32–0.62), most strongly with *Emotional Dysregulation* (*r* = 0.63), *Somatoform Problems* (*r* = 0.61), *Detachment* (*r* = 0.57), *Phobias* (*r* = 0.56), and *Executive & Behavioural Dyscontrol* (*r* = 0.51) and weakest with *Low Sexual Function* (*r* = 0.32) and *Disaffiliative Antagonism* (*r* = 0.32). Greater fear of cancer recurrence was also associated with all psychopathology dimensions in the past cancer subgroup (*p*_BH_ < 0.001), generally with small‐to‐medium effect sizes (ES = 0.15–0.48). Similar to fear of cancer progression, fear of cancer recurrence was most strongly correlated with *Somatoform Problems* (*r* = 0.48), *Phobias* (*r* = 0.39), *Emotional Dysregulation* (*r* = 0.39), *Detachment* (*r* = 0.37), and *Executive & Behavioural Dyscontrol* (*r* = 0.33), and had its weakest association with *Low Appetite & Intake* (*r* = 0.15).

Lastly, in the overall cancer sample, shorter time since cancer diagnosis was associated with higher scores on *Somatoform Problems* (*ρ* = −0.12, *p*_BH_ = 0.013), *Maladaptive Impulse Expression* (*ρ* = −0.10, *p*_BH_ = 0.034), *Emotional Dysregulation* (*ρ* = −0.09, *p*_BH_ = 0.034), *Executive & Behavioural Dyscontrol* (*ρ* = −0.09, *p*_BH_ = 0.034), *Low Appetite & Intake* (*ρ* = −0.09, *p*_BH_ = 0.034), and *Eating Pathology* (*ρ* = −0.09, *p*_BH_ = 0.034). However, these associations were weak, hovering around the threshold typically used to distinguish a small standardised effect size from a negligible effect. When considering the current and past cancer subgroups separately, time since cancer diagnosis was not significantly associated with any psychopathology dimensions after adjusting for multiple‐testing (*p*_BH_ = 0.156–0.924).

## 4 Discussion

This study represents the first systematic application and examination of HiTOP in cancer survivors. Given the substantial potential of HiTOP to enhance clinical practice and research in psycho‐oncology, these findings constitute an important advancement for the field.

### 4.1 Homogeneity and Reliability of the HiTOP‐SR in Cancer Survivors

All HiTOP‐SR scales showed good homogeneity and reliability within our cancer survivor sample. This is fundamental not only to supporting its applicability within this population, but also the ability to use the scales of the measure to develop and examine data‐driven dimensional models of psychopathology in cancer survivors. Given that the HiTOP‐SR has been developed relatively recently, with psychometric properties in different samples still emerging [42, 43, 68], this result provides an important step, marking the first assessment of its homogeneity and reliability in a specific clinical population, beyond psychiatric populations. Further, the use of the HiTOP‐SR facilitated the most comprehensive phenotyping of psychopathology in a cancer survivor population to date; this may be leveraged to inform future priority domains of psychopathology in clinical and research settings.

### 4.2 Detection of Symptom Elevations in Often‐Neglected Domains

As well as showing strong homogeneity and reliability, the HiTOP‐SR displayed the ability to detect elevations in a wide range of psychopathology domains in cancer survivors. Within psycho‐oncology, clinicians and researchers predominantly use tools such as the Distress Thermometer [69] and Hospital Anxiety and Depression Scale [70], which assess components of *Internalising* psychopathology, and do so without providing the required breadth and depth of data to facilitate tailored conceptualisation, treatment planning, and care [6, 7]. There are significant concerns that this narrow assessment focus may miss the identification of elevations among other domains of psychopathology [6, 7].

As expected, the HiTOP‐SR detected elevations within *Internalising* psychopathology across combined current cancer and past cancer samples. However, it also detected significant elevations in *Somatoform* psychopathology. The identification of *Somatoform* psychopathology in cancer survivorship is vital for disentangling the effects that are biologically determined by cancer and its treatments versus those with psychological underpinnings [71]. Despite clinical importance, *Somatoform* psychopathology remains neglected in psycho‐oncology, and DSM criteria have been criticised for limited utility and rigidity [71]. The identification of specific somatoform evaluations without conflating internalising symptoms illustrates the value of a HiTOP‐based approach in this field.

### 4.3 Differential Patterns of Elevated and Sub‐Normative Symptom Severity in Those With Current Versus Past Cancer

Distinguishing people with either current or past cancer further highlighted the HiTOP‐SR's value in detecting often‐overlooked domains in psycho‐oncology. Those currently with cancer showed marked elevations across the *Thought Disorder* spectra, another often‐overlooked area in psycho‐oncology [72, 73]. Those currently with cancer also showed elevations in the often‐neglected assessment spectra of *Disinhibited* and *Antagonistic* psychopathology, which may be linked to a present hedonic and lesser future‐focused orientation [74, 75, 76].

At the finer HiTOP level of Homogeneous Symptom Components and Maladaptive Traits, the HiTOP‐SR identified elevations in domains such as *Health Anxiety, Bodily Distress*, and *Appetite Loss*, patterns expected among individuals currently living with cancer [77, 78, 79]. However, notable elevations also emerged in components such as *Delusions*, another area largely neglected in psycho‐oncology [80]. Conversely, those who had previously had cancer displayed lower levels of *Externalising* and *Thought Disorder* when compared to the normative data. While the literature largely supports the lessening of psychopathology symptoms as individuals progress further across survivorship [81], it was unexpected that those with a past diagnosis of cancer who are now free of disease would display lower levels of symptomology than in normative data. Speculatively, this could reflect post‐traumatic growth [82]. Compared to normative data, lower *Thought Disorder* levels in those who previously had cancer, and higher levels in those currently with cancer, suggest that *Thought Disorder* psychopathology in cancer may be (semi‐)acute stress‐related symptomology (e.g., stress‐induced psychosis) rather than indicative of enduring psychotic illness [83]. Longitudinal data would be needed to assess this hypothesis.

### 4.4 Dimensional Structure of Psychopathology

The 11 factors derived from the analysis of the combined cancer survivor and community/psychiatric samples included factors resembling HiTOP Spectra (i.e., *Somatoform*, *Detachment*, *Disaffiliative Antagonism*), Subfactors (i.e., *Eating Pathology, Low Sexual Function*), Syndromes (i.e., *Low Appetite and Intake, Executive and Behavioural Dyscontrol*), and Homogenous Symptom Components/Maladaptive Traits (i.e., *Phobias, Maladaptive Impulsivity, Emotional Disorganisation, Anankastia*). While some of these factors very closely mirror constructs in the HiTOP structure (i.e., *Somatoform, Eating Pathology, Detachment, Low Sexual Function, and Phobias*), others more loosely resemble HiTOP constructs (i.e., *Executive and Behavioural Dyscontrol, Maladaptive Impulse Expression, Low Appetite and Intake, Emotional Dysregulation, Anankastia*).

While the literature on the dimensional structure and make‐up of HiTOP‐SR is still emerging, the resultant 11‐factors had strong crossover with, but also meaningful differentiation from, existing literature reporting the development of similar models using HiTOP‐SR data in general and psychiatric samples (see [42, 43, 68]). Faulkenberry et al. [42], in their assessment of the structure of the HiTOP‐SR in a community sample, found the lowest‐order factors of *Eating Pathology, Sexual Dysfunction, Anankastia, Thought Disorder, Somatoform & Fear, Negative Affectivity, Suicide, Disinhibited Externalising, Antagonistic Externalising,* and *Narcissism*. The 11‐factor structure resulting from the cancer survivor and community/psychiatric mixed sample structure maps onto these broad domains but differentiates them more finely in some ways, for example, separating somatoform from fears and phobias, broader in other ways, for example, in suicide items not loading onto a separate factor. Similarly, compared with Mostajabi et al.'s [43] analysis of mixed community and psychiatric sample data, which identified the factors of *Psychosis/Rarity, Antagonism, Grandiosity/Dominance, Eating Problems, Fear, Sexual Problems, Risk Taking, Disorganization,* and *Distress*, our cancer‐inclusive sample shows correspondences (e.g., *Eating Pathology, Phobias, Low Sexual Function, Disaffiliative Antagonism*) but again reveals greater granularity in some domains, such as *Somatoform*, and greater breadth in other domains such as *Risk Taking* not forming a separate factor. Relative to Zimmermann et al.'s [44] analysis of data from a community sample, whose factors (not including the substance use module) included, at the lowest level, *Fear/Somatoform Problems, Sexual Problems, Anankastia, Thought Disorder, Hypomania, Body‐Focused Impulse‐Control Problems,* and *Sexual Disinhibition*, the cancer‐inclusive model aligns closely, but again differs from this model in ways such as by the separation of functional domain of somatoform.

The more differentiated structure, particularly within domains such as somatoform, in the cancer‐inclusive sample structure may reflect illness and treatment‐related physiological (e.g., fatigue, pain, metabolic disruption, appetite loss), and psychological (e.g., heightened somatic vigilance, and emotional lability) impacts, which may have sharpened boundaries across somatic, emotional‐regulatory, and impulse‐control processes. However, because the factor structure was derived from the combined cancer survivor and community/psychiatric sample, it remains unclear whether these distinctions are specific to cancer survivorship or would emerge in other heterogeneous samples. Nevertheless, the findings suggest that a more differentiated representation of psychopathology may be useful when assessing mental health in cancer survivors.

### 4.5 Invariance Between Cancer Survivor and Community/Psychiatric Samples

Configural invariance testing provided the strongest support for similarity of structure between cancer survivor and community/psychiatric samples for *Phobias*, *Somatoform Problems*, and *Maladaptive Impulse Expression*. For the remaining dimensions, interpretation was more nuanced because one or more prespecified fit or reliability criteria were not met, although the observed pattern of model misfit was generally similar across groups. This represents the first comparison between these groups for an empirically derived dimensional model of psychopathology and supports the potential applicability of structures such as HiTOP to cancer survivor populations.

The results for three (i.e., *Executive and Behavioural Dyscontrol, Low Sexual Function, Emotional Dysregulation*) of the 11 factors further suggested that each scale within these respective domains related to its underlying factor with similar strength in both samples. The absence of clear evidence for configural or metric non‐invariance across samples suggests that the factors, *Executive and Behavioural Dyscontrol, Low Sexual Function,* and *Emotional Dysregulation*, can be interpreted consistently between cancer survivors and non‐cancer‐specific groups. The remaining eight factors (i.e., *Phobias, Somatoform, Eating Pathology, Low Appetite and Intake, Maladaptive Symptom Expression, Detachment, Disaffiliative Antagonism, Anankastia*), however, showed evidence of metric non‐invariance, suggesting that the loading of one or more items within each factor differed between the groups. Differences in item loadings within these factors across samples may reflect cancer‐specific experiences and effects, as well as symptom and personality traits linked to behaviours that increase cancer risk (e.g., smoking). For example, cancer‐related experiences, such as chemotherapy and surgery, may account for the heightened loading of the Blood‐Injection Phobia scale on the *Phobias* factor among cancer survivors. Further, the weaker loading of Risk Aversion and greater loading of Perfectionism on the *Anankastia* factor may be reflective of the greater tendency for risky behaviours, such as smoking, among people who are diagnosed with cancer, and the strong prevalence of clinically significant maladaptive perfectionism (up to 1 in 2) in cancer survivors, which is theoretically related to ongoing uncertainty and related coping strategies [84, 85, 86]. Thus, Risk‐Aversion may be less indicative of *Anankastia* in cancer survivors, and Perfectionism more indicative, when compared to community/psychiatric populations.

Overall, the configural and metric non‐invariance findings suggest that while a HiTOP‐SR‐derived dimensional model of psychopathology can be represented and applied in a similar manner between cancer and community/psychiatric populations, additional care must be taken in the interpretation of scores for cancer survivors. Specifically, clinicians and researchers may utilise an understanding of cancer‐related experiences, effects, and common psychological characteristics to provide additional context to evaluations among factors. Some factors derived from HiTOP measures may require additional item‐level examination for cancer survivors to inform tailored and person‐level clinical care and fine‐grained research.

### 4.6 The Strong External Validity of the Dimensional Model in Cancer Survivors

The external validity of the 11‐factor dimensional model of psychopathology within cancer survivors was strong, showing meaningful and theoretically aligned associations between psychological, physical and clinical history domains that are a priority in cancer survivorship clinical practice and research. In line with the existing literature [87], poorer physical functioning was associated with greater psychopathology across the factors, with the *Somatoform* factor displaying the strongest association. Further, as expected, greater fear of cancer recurrence and fear of cancer progression were most strongly associated with factors such as *Somatoform*, *Phobias*, and *Emotional Dysregulation*. While these associations may be theoretically anticipated, their examination provides an important test of whether prominent psycho‐oncology constructs map onto the HiTOP dimensions in a coherent manner, thereby supporting the external validity and clinical interpretability of the resulting psychopathology structure. Both fear of cancer recurrence and fear of cancer progression may be expected to associate strongly with *Somatoform* symptomology, reflecting heightened personal cancer symptom awareness and bodily monitoring [88]. Fear of cancer recurrence and fear of cancer progression may also be expected to correlate strongly with factors such as *Phobias* and *Emotional Dysregulation*, due to their shared higher‐order psychopathology, which may be interpreted as representing *Internalising* psychopathology.

Ultimately, in addition to being applicable to cancer survivors, the 11‐factor dimensional model of psychopathology showed the expected convergence with cancer‐relevant physical and psychological characteristics. These findings highlight the applicability and utility of adopting dimensional models in cancer survivorship research and practice, offering a framework that captures the complexity of psychopathology and paving the way for more precise, personalised, and integrated care.

### 4.7 Strengths, Limitations, and Directions for Future Research

This research had several significant strengths. First, it utilised a large sample (*N* = 1389 plus an *n* = 780 HiTOP‐SR normative sample), which included the most comprehensive phenotyping of psychopathology in a cancer survivor population to date. Second, the cancer survivor sample included both those currently with cancer and those who previously had cancer, and included a wide range of cancer types, stages, treatments received, and time since diagnosis. Third, the community/psychiatric sample utilised those from the general population as well as those seeking treatment, facilitating a broad spectrum of symptom severities. Finally, rigorous analytical approaches, informed by best practice principles and recommendations [51, 52, 53, 63, 64, 67], were used.

Although this research had a number of key strengths, it also had limitations. First, cancer history and all clinical data were self‐reported. Second, participants were recruited through an online platform, which may have introduced selection bias. In particular, cancer survivors who are older, have lower digital literacy, or experience greater medical comorbidity may have been underrepresented. Furthermore, the sample was drawn predominantly from English‐speaking participants residing in the United States and United Kingdom. Consequently, the extent to which these findings generalise to culturally and linguistically diverse populations remains to be established. Third, given the ongoing development, the HiTOP‐SR substance use module could not be included. Future research should administer the HiTOP‐SR, inclusive of the substance‐use module. Fourth, there were notable demographic differences in age, sex, and country of residence. Consequently, while the invariance testing and other results were interpreted primarily from the lens of cancer‐specific effects and experiences, group differences cannot be solely attributed to cancer survivorship status and may have been influenced by demographic factors. Future research should seek to replicate these findings using demographically matched samples. Nevertheless, previous research has generally supported comparable HiTOP structures across major demographic groups [89, 90]. Whether these findings extend to cancer survivor populations, and particularly to intersections between demographic and cancer‐related factors, is currently known. Relatedly, the dimensional structure was derived from the combined cancer survivor and community/psychiatric sample. Consequently, although the resulting factor solution demonstrated relevance and utility within the cancer survivor sample, it cannot be assumed that features of the structure are unique to cancer survivorship. Further, due to convergence issues when fitting the full dimensional model, invariance was evaluated one dimension at a time rather than across the full intercorrelated structure. Consequently, these analyses should be interpreted as a partial test of invariance. Lastly, in line with related assessments [43], a single‐level dimensional model was developed and assessed, rather than a full hierarchical model. The most fine‐grained factor solution was focused upon, as it is likely to be of greatest interest to clinicians. However, future research should develop and evaluate a full hierarchical model of psychopathology in cancer survivors and conduct these evaluations using age‐ and sex‐matched samples. Future research should also assess the reliability and validity of the brief version of the HiTOP‐SR, the HiTOP‐Brief (B‐HiTOP) [32], which provides an assessment of the higher‐order spectra of the HiTOP model. The B‐HiTOP, designed for greater clinical feasibility, must be tested in cancer survivor samples to assist in clinical translation. To further facilitate clinical translation, an empirical assessment of the clinical utility of HiTOP in psycho‐oncology, directly compared to the DSM and ICD, must also be conducted [15, 91]. Finally, the findings of this research should inform the adaptation and refinement of existing HiTOP training for clinicians to support its application within psycho‐oncology.

### 4.8 Implications (Clinical and Research)

These findings have important implications for both clinical practice and research in psycho‐oncology. Clinically, the demonstrated reliability, ability to detect meaningful symptom elevations, and external validity of the HiTOP in cancer survivors support the use of dimensional models to meaningfully and precisely characterise psychological challenges. Notably, elevations were observed across multiple psychopathology domains in people currently with cancer, including those not routinely assessed in psycho‐oncology (e.g., manic energy, risk‐taking, delusions), suggesting that current approaches may overlook clinically relevant difficulties. A HiTOP‐informed approach may improve case formulation by differentiating overlapping symptom domains, particularly internalising, somatoform, and identifying meaningful elevations in less routinely assessed domains such as thought disorder and externalising, thereby supporting more tailored, person‐centred interventions.

From a research perspective, these results provide a foundation for integrating dimensional psychopathology frameworks into psycho‐oncology research, enabling more comprehensive phenotyping and improved alignment between psychological, physical, and cancer‐specific outcomes. Further, this approach facilitates a more nuanced exploration of the aetiology of psychopathology in cancer survivors, and more robust and comprehensive outcome assessment approaches in clinical trials. The applicability of HiTOP to cancer also provides a foundation for psycho‐oncology research to interface with comparative contemporary research in other populations.

## 5 Conclusion

The HiTOP‐SR appears reliable among cancer survivors. The measure also provided utility in identifying a of broad elevations across multiple domains of psychopathology, including those not routinely assessed or explored in psycho‐oncology clinical practice and research. This underscores that HiTOP may facilitate more compressive and holistic clinical case‐formulations and research explorations in psycho‐oncology. The results support the applicability and utility of dimensional models such as HiTOP for both clinical practice and research in psycho‐oncology, while also highlighting the importance of considering context, like cancer‐related experiences, treatment effects, and individual characteristics, in interpreting measures of these models. Finally, this research supports the alignment between the HiTOP‐derived factors and physical and psychological characteristics of particular relevance to cancer. Ultimately, dimensional models of psychopathology have strong potential to advance clinical practice and research in psycho‐oncology.

## Author Contributions

**Darren Haywood:** conceptualization, methodology, writing – original draft. **Jai Carmichael:** methodology, formal analysis, writing – original draft. **Miriam K. Forbes:** methodology, writing – review and editing. **Roman Kotov:** methodology, writing – review and editing. **Robert F. Krueger:** methodology, writing – review and editing. **Aidan G. C. Wright:** methodology, writing – review and editing. **Susan L. Rossell:** methodology, writing – review and editing. **David Castle:** writing – review and editing. **Wendy Lam:** writing – review and editing. **Maryam Lustberg:** writing – review and editing. **Colin E. Vize:** writing – review and editing. **Anna Ugalde:** writing – review and editing. **Phyllis Butow:** writing – review and editing. **Nicolas H. Hart:** methodology, writing – review and editing. All authors approved the final version of the manuscript.

## Funding

This research was supported by a UTS Seed Grant. DH receives salary support from the Cancer Institute NSW as an Early Career Investigator Fellow (2025/ECF2620) and the University of Technology Sydney as a Chancellors Research Fellow (PRO25‐23000). NHH and MKF receive salary support from the National Health and Medical Research Council (NHMRC) as Investigator Fellows (APP2017080 and APP1194292). SLR was funded by a Senior NHMRC Fellowship (GNT1154651). AGCW is supported by the National Institutes of Health (R01 AA026879, R01 MH048463, R01 MH131606). RFK was supported partly by National Institutes of Health grants R01AG053217, R01AG077742, and U19AG51426. CEV is supported by the National Institutes of Health (K01 MH130746). AU is supported by a Cancer Research Fellowship, Victoria.

## Conflicts of Interest

D.H., M.K.F., R.K., R.F.K., C.E.V., and A.C.G.W. are members of the HiTOP Consortium. M.K.F. is HiTOP President, M.K.F., R.K., R.F.K., and A.G.C.W. are Executive Committee members of HiTOP.

## Supporting information


Supporting Information S1

## Data Availability

The data that support the findings of this study are available on request from the corresponding author. The data are not publicly available due to privacy or ethical restrictions.

## References

[1] D. Haywood , A. Chan , M. B. Lustberg , et al., “Planning for Cancer: Building Accessible and High‐Quality Survivorship Care for all,” Trends in Cancer 12, no. 4 (2026): 320–327, https://www.cell.com/trends/cancer/abstract/S2405‐8033(25)00313‐9. 41483982 10.1016/j.trecan.2025.12.006

[2] S. Kuhnt , E. Brähler , H. Faller , et al., “Twelve‐Month and Lifetime Prevalence of Mental Disorders in Cancer Patients,” Psychotherapy and Psychosomatics 85, no. 5 (2016): 289–296, 10.1159/000446991. 27508418

[3] G. Joshy , J. Thandrayen , B. Koczwara , et al., “Disability, Psychological Distress and Quality of Life in Relation to Cancer Diagnosis and Cancer Type: Population‐Based Australian Study of 22,505 Cancer Survivors and 244,000 People Without Cancer,” BMC Medicine 18 (2020): 1–15, 10.1186/s12916-020-01830-4. 33256726 PMC7708114

[4] Z. Steel , C. Marnane , C. Iranpour , et al., “The Global Prevalence of Common Mental Disorders: A Systematic Review and Meta‐Analysis 1980–2013,” International Journal of Epidemiology 43, no. 2 (2014): 476–493, 10.1093/ije/dyu038. 24648481 PMC3997379

[5] C. E. Low , S. Loke , G. E. Pang , B. Sim , and V. S. Yang , “Psychological Outcomes in Patients With Rare Cancers: A Systematic Review and meta‐analysis,” EClinicalMedicine 72 (2024): 102631, 10.1016/j.eclinm.2024.102631. 38726223 PMC11079476

[6] D. Haywood , R. Kotov , R. F. Krueger , et al., “Is it Time to Discard the Diagnostic and Statistical Manual of Mental Disorders (DSM) in Psycho‐Oncology?,” Cancer Letters 589 (2024): 216818, 10.1016/j.canlet.2024.216818. 38554804

[7] D. Haywood , R. Kotov , R. F. Krueger , et al., “Reconceptualizing Mental Health in Cancer Survivorship,” Trends in Cancer 10, no. 8 (2024): 677–686, 10.1016/j.trecan.2024.05.008. 38890021

[8] S. Chen , Z. Cao , K. Prettner , et al., “Estimates and Projections of the Global Economic Cost of 29 Cancers in 204 Countries and Territories From 2020 to 2050,” JAMA Oncology 9, no. 4 (2023): 465–472, 10.1001/jamaoncol.2022.7826. 36821107 PMC9951101

[9] Cancer Australia , Australian Cancer Plan (Cancer Australia, 2023), https://www.australiancancerplan.gov.au/.

[10] National Cancer Institute , “National Cancer Plan,” (2024), https://cancercontrol.cancer.gov/national‐cancer‐plan.

[11] European Commission , “Europe’s Beating Cancer Plan,” (2021), https://health.ec.europa.eu/system/files/2022‐02/eu_cancer‐plan_en_0.pdf.

[12] E. Gilboa‐Schechtman , “Case Conceptualization in Clinical Practice and Training,” supplement, Clinical Psychology in Europe 6, no. S (2024): e12103, 10.32872/cpe.12103. 39118655 PMC11303933

[13] D. Haywood , D. J. Castle , and N. H. Hart , “Avoiding the Pitfalls of the DSM‐5: A Primer for Health Professionals,” General Hospital Psychiatry 90 (2024): 88–90, 10.1016/j.genhosppsych.2024.07.006. 39053381

[14] M. J. Cordova , M. B. Riba , and D. Spiegel , “Post‐Traumatic Stress Disorder and Cancer,” Lancet Psychiatry 4, no. 4 (2017): 330–338, 10.1016/s2215-0366(17)30014-7. 28109647 PMC5676567

[15] C. E. Balling , S. C. South , D. R. Lynam , and D. B. Samuel , “Clinician Perception of the Clinical Utility of the Hierarchical Taxonomy of Psychopathology (HiTOP) System,” Clinical Psychological Science (2023): 21677026221138818.

[16] M. B. First , T. J. Rebello , J. W. Keeley , et al., “Do Mental Health Professionals Use Diagnostic Classifications the Way We Think They Do? A Global Survey,” World Psychiatry 17, no. 2 (June 2018): 187–195, 10.1002/wps.20525. 29856559 PMC5980454

[17] S. C. Evans , G. M. Reed , M. C. Roberts , et al., “Psychologists' Perspectives on the Diagnostic Classification of Mental Disorders: Results From the WHO‐IUPsyS Global Survey,” International Journal of Psychology 48, no. 3 (2013): 177–193, 10.1080/00207594.2013.804189. 23750927 PMC3725658

[18] C. R. Ridley , C. E. Jeffrey , and R. B. Roberson Iii , “Case Mis‐Conceptualization in Psychological Treatment: An Enduring Clinical Problem,” Journal of Clinical Psychology 73, no. 4 (2017): 359–375, 10.1002/jclp.22354. 28085194

[19] M. A. Waszczuk , M. Zimmerman , C. Ruggero , et al., “What Do Clinicians Treat: Diagnoses or Symptoms? The Incremental Validity of a Symptom‐Based, Dimensional Characterization of Emotional Disorders in Predicting Medication Prescription Patterns,” Comprehensive Psychiatry 79 (2017): 80–88, 10.1016/j.comppsych.2017.04.004. 28495012 PMC5643213

[20] J. Carmichael and D. Haywood , “For Clinical Translation, HiTOP Must Stand on Its Own Two Feet,” Journal of Psychopathology and Clinical Science 134, no. 5 (2025): 486–487, 10.1037/abn0001004. 40310187

[21] L. Grassi , R. Caruso , S. Sabato , S. Massarenti , and M. G. Nanni , “Psychosocial Screening and Assessment in Oncology and Palliative Care Settings,” Frontiers in Psychology 5 (2015): 1485, 10.3389/fpsyg.2014.01485. 25709584 PMC4285729

[22] R. Kotov , R. F. Krueger , D. Watson , et al., “The Hierarchical Taxonomy of Psychopathology (HiTOP): A Quantitative Nosology Based on Consensus of Evidence,” Annual Review of Clinical Psychology 17, no. 1 (2021): 83–108, 10.1146/annurev-clinpsy-081219-093304. 33577350

[23] D. Haywood , F. D. Baughman , B. A. Mullan , and K. R. Heslop , “Psychopathology and Neurocognition in the Era of the p‐Factor: The Current Landscape and the Road Forward,” Psychiatry International 2, no. 3 (2021): 233–249, 10.3390/psychiatryint2030018.

[24] R. Kotov , R. F. Krueger , D. Watson , et al., “The Hierarchical Taxonomy of Psychopathology (HiTOP): A Dimensional Alternative to Traditional Nosologies,” Journal of Abnormal Psychology 126, no. 4 (2017): 454–477, 10.1037/abn0000258. 28333488

[25] R. Kotov , W. T. Carpenter , D. C. Cicero , et al., “Psychosis Superspectrum II: Neurobiology, Treatment, and Implications,” Molecular Psychiatry 29, no. 5 (2024): 1–17, 10.1038/s41380-024-02410-1. 38351173 PMC11731826

[26] R. Kotov , D. C. Cicero , C. C. Conway , et al., “The Hierarchical Taxonomy of Psychopathology (HiTOP) in Psychiatric Practice and Research,” Psychological Medicine 52, no. 9 (2022): 1666–1678, 10.1017/s0033291722001301. 35650658

[27] L. J. Simms , A. G. C. Wright , D. Cicero , et al., “Development of Measures for the Hierarchical Taxonomy of Psychopathology (HiTOP): A Collaborative Scale Development Project,” Assessment 29, no. 1 (2022): 3–16, 10.1177/10731911211015309. 34013772

[28] S. Cheli , W. W. T. Lam , T. Estapé , et al., “Risk Perception, Treatment Adherence, and Personality During COVID‐19 Pandemic: An International Study on Cancer Patients,” Psycho‐Oncology 31, no. 1 (2022): 46–53, 10.1002/pon.5775. 34314560 PMC8420575

[29] S. Cheli , M. S. Pino , G. Goldzweig , et al., “The Relationship Between COVID‐19 Risk Perception and Vaccine Hesitancy in Cancer Patients: The Moderating Role of Externalizing Traits,” Clinical Neuropsychiatry 19, no. 6 (2022): 355, 10.36131/cnfioritieditore20220602. 36627943 PMC9807116

[30] Cancer Australia , “Optimal Care Pathway for Older People With Cancer,” (2025), https://www.cosa.org.au/advocacy/ocp‐for‐older‐people‐with‐cancer/.

[31] Clinical Oncology Society of Australia , “Guidelines for the Psychosocial Management of Adolescents and Young Adults Diagnosed With Cancer,” (2025), https://www.cancer.org.au/clinical‐guidelines/adolescents‐young‐adults/psychosocial‐management‐of‐ayas‐diagnosed‐with‐cancer.

[32] “Hierarchical Taxonomy of Psychopathology Society, the HiTOP self‐report Measures,” (2026), https://www.hitop‐system.org/hitop‐self‐report‐measures.

[33] S. Palan and C. Schitter , “Prolific.ac—A Subject Pool for Online Experiments,” Journal of Behavioral and Experimental Finance 17 (2018): 22–27, 10.1016/j.jbef.2017.12.004.

[34] K. Uittenhove , S. Jeanneret , and E. Vergauwe , “From Lab‐Based to Web‐Based Behavioural Research: Who You Test Is More Important than How You Test,” Journal of Cognition 6 (2023): 13, 10.31234/osf.io/uy4kb. 36721797 PMC9854315

[35] B. D. Douglas , P. J. Ewell , and M. Brauer , “Data Quality in Online Human‐Subjects Research: Comparisons Between MTurk, Prolific, CloudResearch, Qualtrics, and SONA,” PLoS One 18, no. 3 (2023): e0279720, 10.1371/journal.pone.0279720. 36917576 PMC10013894

[36] D. A. Albert and D. Smilek , “Comparing Attentional Disengagement Between Prolific and MTurk Samples,” Scientific Reports 13, no. 1 (2023): 20574, 10.1038/s41598-023-46048-5. 37996446 PMC10667324

[37] E. Peer , D. Rothschild , A. Gordon , Z. Evernden , and E. Damer , “Data Quality of Platforms and Panels for Online Behavioral Research,” Behavior Research Methods 54, no. 4 (2022): 1643–1662, 10.3758/s13428-021-01694-3. 34590289 PMC8480459

[38] D. Haywood , A. Chan , R. J. Chan , et al., “The MASCC COG‐IMPACT: An Unmet Needs Assessment for Cancer‐Related Cognitive Impairment Impact Developed by the Multinational Association of Supportive Care in Cancer,” Supportive Care in Cancer 33, no. 2 (2025): 120, 10.1007/s00520-025-09149-7. 39853439 PMC11761510

[39] D. Haywood , F. Baughman , B. Mullan , and K. Heslop , “What Accounts for the Factors of Psychopathology? An Investigation of the Neurocognitive Correlates of Internalising, Externalising, and the p‐Factor,” Brain Sciences 12, no. 4 (2022): 421, 10.3390/brainsci12040421. 35447951 PMC9030002

[40] W. R. Ringwald , G. King , C. E. Vize , and A. G. C. Wright , “Passive Smartphone Sensors for Detecting Psychopathology,” JAMA Network Open 8, no. 7 (2025): e2519047, 10.1001/jamanetworkopen.2025.19047. 40608335 PMC12232220

[41] WHO , “Global Cancer Burden Growing, Amidst Mounting Need for Services,” (2024), https://www.who.int/news/item/01‐02‐2024‐global‐cancer‐burden‐growing‐amidst‐mounting‐need‐for‐services. PMC11115397 38438207

[42] R. S. Faulkenberry , K. Y. Lee , R. J. Linscott , et al., “Validation of the Hierarchical Taxonomy of Psychopathology–Self Report (HiTOP‐SR): Internal Structure and Construct Validity Against the MMPI‐3 in a Community Sample,” Open Science Framework (2025), https://osf.io/download/nqwe3. 10.1037/pas0001470 42080896

[43] J. Mostajabi , C. Vize , S. Nielsen , W. R. Ringwald , and A. G. C. Wright , “Structure of Current Psychopathology and Its Associations With Daily Life Experiences Using the HiTOP‐PRO in a Mixed Clinical/Community Sample,” Open Science Framework 38, no. 6–7 (2025): 451–465, 10.1037/pas0001455. PMC13489107 41746693

[44] J. Zimmermann , L. P. Wendt , H. Edelhoff , et al., “Development and Initial Evaluation of the German Version of the Hierarchical Taxonomy of Psychopathology Self‐Report (HiTOP‐SR),” Journal of Psychopathology and Clinical Science (2024), Advance online publication.

[45] A. B. Smith , J. E. Fardell , and P. N. Butow , “Fear of Cancer Recurrence,” in Psycho‐Oncology, ed. P. N. Butow , W. W. T. Lam , P. N. Butow , et al. (Oxford University Press, 2021).

[46] A. B. Smith , M. Gao , M. Tran , et al., “Evaluation of the Validity and Screening Performance of a Revised Single‐Item Fear of Cancer Recurrence Screening Measure (FCR‐1r),” Psycho‐Oncology 32, no. 6 (2023): 961–971, 10.1002/pon.6139. 37120796

[47] Y. Youssef , A. Mehnert‐Theuerkauf , H. Götze , M. Friedrich , and P. Esser , “Rapid Screener for the Assessment of Fear of Progression in Cancer Survivors: The Fear of Progression‐Questionnaire Rapid Screener,” European Journal of Cancer Care 30, no. 3 (2021): e13400, 10.1111/ecc.13400. 33459435

[48] J. E. Ware , SF‐36 Health Survey. Manual and Interpretation Guide (Health Institute, 1993), 6–17.

[49] Y. Rosseel , “Lavaan: An R Package for Structural Equation Modeling,” Journal of Statistical Software 48, no. 1 (2012): 1–36, 10.18637/jss.v048.i02.

[50] W. Revelle , Psych: Procedures for Psychological, Psychometric, and Personality Research (Northwestern University, 2024), https://CRAN.R‐project.org/package=psych.

[51] D. Shi , A. Maydeu‐Olivares , and C. DiStefano , “The Relationship Between the Standardized Root Mean Square Residual and Model Misspecification in Factor Analysis Models,” Multivariate Behavioral Research 53, no. 5 (2018): 676–694, 10.1080/00273171.2018.1476221. 30596259

[52] C. Ximénez , A. Maydeu‐Olivares , D. Shi , and J. Revuelta , “Assessing Cutoff Values of SEM Fit Indices: Advantages of the Unbiased SRMR Index and Its Cutoff Criterion Based on Communality,” Structural Equation Modeling: A Multidisciplinary Journal 29, no. 3 (2022): 368–380, 10.1080/10705511.2021.1992596.

[53] M. K. Forbes , A. L. Greene , H. F. Levin‐Aspenson , et al., “Three Recommendations Based on a Comparison of the Reliability and Validity of the Predominant Models Used in Research on the Empirical Structure of Psychopathology,” Journal of Abnormal Psychology 130, no. 3 (2021): 297–317, 10.1037/abn0000533. 33539117

[54] W. Revelle and R. E. Zinbarg , “Coefficients Alpha, Beta, Omega, and the Glb: Comments on Sijtsma,” Psychometrika 74, no. 1 (2009): 145–154, 10.1007/s11336-008-9102-z.

[55] R. P. McDonald , Test Theory: A Unified Approach (Erlbaum, 1999).

[56] R. E. Zinbarg , W. Revelle , I. Yovel , and W. Li , “Cronbach’s α, Revelle’s β, and Mcdonald’s ωH: Their Relations With Each Other and Two Alternative Conceptualizations of Reliability,” Psychometrika 70, no. 1 (2005): 123–133, 10.1007/s11336-003-0974-7.

[57] B. P. O’connor , “SPSS and SAS Programs for Determining the Number of Components Using Parallel Analysis and Velicer’s MAP Test,” Behavior Research Methods, Instruments, & Computers 32, no. 3 (2000): 396–402, 10.3758/bf03200807. 11029811

[58] W. F. Velicer , “Determining the Number of Components From the Matrix of Partial Correlations,” Psychometrika 41, no. 3 (1976): 321–327, 10.1007/bf02293557.

[59] J. Carmichael , J. Ponsford , K. R. Gould , et al., “A Transdiagnostic, Hierarchical Taxonomy of Psychopathology Following Traumatic Brain Injury (HiTOP‐TBI),” Journal of Neurotrauma 42, no. 7–8 (2025): 714–730, 10.1089/neu.2024.0006. 38970424

[60] M. Forbes , T. Pham , M. Roberts , and C. Johnco , “Rebuilding HiTOP From the Ground Up: Symptom‐Level Analyses and a Revised Mapping to the DSM,” Open Science Framework (2025), https://osf.io/preprints/psyarxiv/8tm6c. 10.1037/abn0001125 42080848

[61] M. K. Forbes , A. Baillie , P. J. Batterham , et al., “Reconstructing Psychopathology: A Data‐Driven Reorganization of the Symptoms in the Diagnostic and Statistical Manual of Mental Disorders,” Clinical Psychological Science 13, no. 3 (2025): 462–488, 10.1177/21677026241268345. 41415680 PMC12711323

[62] M. K. Forbes , A. L. Watts , M. Twose , et al., “A Hierarchical Model of the Symptom‐Level Structure of Psychopathology in Youth,” Clinical Psychological Science 13, no. 2 (2025): 278–300, 10.1177/21677026241257852. PMC12054596 40330987

[63] R. Luong and J. K. Flake , “Measurement Invariance Testing Using Confirmatory Factor Analysis and Alignment Optimization: A Tutorial for Transparent Analysis Planning and Reporting,” Psychological Methods 28, no. 4 (2023): 905–924, 10.1037/met0000441. 35588078

[64] P. J. Ferrando and U. Lorenzo‐Seva , “Assessing the Quality and Appropriateness of Factor Solutions and Factor Score Estimates in Exploratory Item Factor Analysis,” Educational and Psychological Measurement 78, no. 5 (2018): 762–780, 10.1177/0013164417719308. 32655169 PMC7328234

[65] R. L. Gorsuch , Factor Analysis, 2 ed. (Lawrence Erlbaum Associates, 1983).

[66] J. W. Grice , “Computing and Evaluating Factor Scores,” Psychological Methods 6, no. 4 (2001): 430–450, 10.1037/1082-989x.6.4.430. 11778682

[67] A. Rodriguez , S. P. Reise , and M. G. Haviland , “Applying Bifactor Statistical Indices in the Evaluation of Psychological Measures,” Journal of Personality Assessment 98, no. 3 (2016): 223–237, 10.1080/00223891.2015.1089249. 26514921

[68] W. Zhang , P. Wang , R. Liu , et al., “Validation of the Mandarin Chinese Version of the Detachment, Internalizing, and Somatoform Spectra of the Hierarchical Taxonomy of Psychopathology Self‐Report (HiTOP‐SR),” Open Science Framework (2025), https://osf.io/download/t6vxy.

[69] K. A. Donovan , L. Grassi , H. L. McGinty , and P. B. Jacobsen , “Validation of the Distress Thermometer Worldwide: State of the Science,” Psycho‐Oncology 23, no. 3 (2014): 241–250, 10.1002/pon.3430. 25160838

[70] I. Bjelland , A. A. Dahl , T. T. Haug , and D. Neckelmann , “The Validity of the Hospital Anxiety and Depression Scale: An Updated Literature Review,” Journal of Psychosomatic Research 52, no. 2 (2002): 69–77, 10.1016/s0022-3999(01)00296-3. 11832252

[71] L. Grassi , R. Caruso , and M. G. Nanni , “Somatization and Somatic Symptom Presentation in Cancer: A Neglected Area,” International Review of Psychiatry 25, no. 1 (2013): 41–51, 10.3109/09540261.2012.731384. 23383666

[72] J. Bergqvist , S. Hedskog , C. Hedman , T. Schultz , and P. Strang , “Patients With Both Cancer and Psychosis—To What Extent Do They Receive Specialized Palliative Care,” Acta Psychiatrica Scandinavica 149, no. 4 (2024): 313–322, 10.1111/acps.13666. 38369614

[73] D. Pettersson , M. Gissler , J. Hällgren , U. Ösby , J. Westman , and W. Bobo , “The Overall and Sex‐and Age‐Group Specific Incidence Rates of Cancer in People With Schizophrenia: A Population‐Based Cohort Study,” Epidemiology and Psychiatric Sciences 29 (2020): e132, 10.1017/s204579602000044x. 32460950 PMC7264860

[74] M. Moskalewicz , P. Kordel , and A. Sterna , “The Rhythm of Chemotherapy and Cancer Patients’ Time Perspectives,” PeerJ 10 (2022): e14486, 10.7717/peerj.14486. 36536628 PMC9758969

[75] F. Andreis , M. Mirandola , A. C. Wedenissow , et al., “Dignity and Time Perspective: A Pilot Explorative Study in Cancer Patients,” Palliative & Supportive Care 21, no. 1 (2023): 43–48, 10.1017/s1478951522000402. 35393000

[76] T. Wang , Y. Guo , K. Zhao , C. Tang , and Q. Xu , “The Relationship Between Time Perspective and Fear of Cancer Recurrence Among Chinese Gastric Cancer Patients: The Chain Mediating Role of Rumination and Catastrophizing,” Supportive Care in Cancer 33, no. 4 (2025): 271, 10.1007/s00520-025-09342-8. 40072738

[77] C. B. Harrington , J. A. Hansen , M. Moskowitz , B. L. Todd , and M. Feuerstein , “It's Not Over When It's Over: Long‐Term Symptoms in Cancer Survivors—A Systematic Review,” International Journal of Psychiatry in Medicine 40, no. 2 (2010): 163–181, 10.2190/pm.40.2.c. 20848873

[78] D. E. B. Galindo , A. Vidal‐Casariego , A. Calleja‐Fernández , et al., “Appetite Disorders in Cancer Patients: Impact on Nutritional Status and Quality of Life,” Appetite 114 (2017): 23–27, 10.1016/j.appet.2017.03.020. 28315777

[79] C. Maheu , M. Singh , W. L. Tock , et al., “Fear of Cancer Recurrence, Health Anxiety, Worry, and Uncertainty: A Scoping Review About Their Conceptualization and Measurement Within Breast Cancer Survivorship Research,” Frontiers in Psychology 12 (2021): 644932, 10.3389/fpsyg.2021.644932. 33912113 PMC8072115

[80] L. Grassi , “Psychiatric and Psychosocial Implications in Cancer Care: The Agenda of Psycho‐Oncology,” Epidemiology and Psychiatric Sciences 29 (2020): e89, 10.1017/s2045796019000829. 31915101 PMC7214699

[81] H. Forbes , H. Carreira , G. Funston , et al., “Early, Medium and Long‐Term Mental Health in Cancer Survivors Compared With Cancer‐Free Comparators: Matched Cohort Study Using Linked UK Electronic Health Records,” eClinicalMedicine 76 (2024): 102826, 10.1016/j.eclinm.2024.102826. 39318789 PMC11421364

[82] P. Blickle , M. E. Schmidt , and K. Steindorf , “Post‐Traumatic Growth in Cancer Survivors: What Is Its Extent and What Are Important Determinants?,” International Journal of Clinical and Health Psychology 24, no. 1 (2024): 100418, 10.1016/j.ijchp.2023.100418. 37867603 PMC10585376

[83] V. Mondelli , “From Stress to Psychosis: Whom, How, When and Why?,” Epidemiology and Psychiatric Sciences 23, no. 3 (2014): 215–218, 10.1017/s204579601400033x. 24905592 PMC6998263

[84] S. El‐din F. Abdallah , I. S. Ramadan , and S. Salah Elsayed , “Relation Between Self‐Compassion, Perfectionism and Body Image Satisfaction Among Women With Mastectomy,” Egyptian Journal of Health Care 12, no. 4 (2021): 1902–1913, 10.21608/ejhc.2021.307158.

[85] C. Trudel‐Fitzgerald , J. Savard , L.‐M. Slim , et al., “The Relationship of Perfectionism With Psychological Symptoms in Cancer Patients and the Contributing Role of Hyperarousability and Coping,” Psychology and Health 32, no. 4 (April 2017): 381–401, 10.1080/08870446.2016.1273354. 28097876

[86] M. Blom , O. R. Guicherit , and M. T. Hoogwegt , “Perfectionism, Intolerance of Uncertainty and Coping in Relation to Fear of Cancer Recurrence in Breast Cancer Patients,” Psycho‐Oncology 32, no. 4 (April 2023): 581–588, 10.1002/pon.6102. 36702980

[87] Y. Zhang , J. Thandrayen , K. Soga , et al., “Physical Disability and Psychological Distress Before and After a Diagnosis of Cancer: Evidence on Multiple Cancer Types From a Large Australian Cohort Study, Compared to People Without a Cancer Diagnosis,” BMC Medicine 23, no. 1 (2025): 290, 10.1186/s12916-025-04111-0. 40389978 PMC12090494

[88] S. Bitan , S. Daches , and I. Hasson‐Ohayon , “Somatosensory Amplification and Psychological Distress in Cancer Survivors: The Mediating Role of Fear of Cancer Recurrence,” Health Psychology and Behavioral Medicine 13, no. 1 (2025): 2525184, 10.1080/21642850.2025.2525184. 40606957 PMC12217098

[89] C. Rodriguez‐Seijas , J. J. Li , C. Balling , et al., “Diversity and the Hierarchical Taxonomy of Psychopathology (HiTOP),” Nature Reviews Psychology 2, no. 8 (2023): 1–13, 10.1038/s44159-023-00200-0.

[90] D. C. Cicero , C. J. Ruggero , C. E. Balling , et al., “State of the Science: The Hierarchical Taxonomy of Psychopathology (HiTOP),” Behavior Therapy 55, no. 6 (2024): 1114–1129, 10.1016/j.beth.2024.05.001. 39443056

[91] C. Hetfeld , K. B. Schmitt , A.‐K. Bräscher , and M. Witthöft , “Clinical Utility and Applicability of the Hierarchical Taxonomy of Psychopathology (HiTOP) vs. ICD‐11: A Comparative Analysis,” Open Science Framework (2025), https://osf.io/download/2rzup.

