# Supplementary material for: Advancing Dimensional Models of Psychopathology in Cancer: Insights From Applying the Hierarchical Taxonomy of Psychopathology (HiTOP)
Source: Psychooncology. 2026 Jun 26;35(7):e70533. doi: 10.1002/pon.70533 (PMC13307643; doi:10.1002/pon.70533)
Supplement: Supplementary file 1 — Supporting Information S1 [file PON-35-e70533-s001.docx]

**Supplemental Material for “ADVANCING DIMENSIONAL MODELS OF PSYCHOPATHOLOGY IN CANCER: INSIGHTS FROM APPLYING THE HIERARCHICAL TAXONOMY OF PSYCHOPATHOLOGY (HITOP)”**

**Supplemental Table S1** *Modified Versions of the HiTOP-SR For Combined Analyses Relying on Both the Cancer-Survivor and Psychiatric/Community Samples*

| **HiTOP-SR scale** | **Modification for combined analyses** |
| --- | --- |
| Cleaning | Entire scale excluded from combined analyses as not administered in psychiatric/community sample. |
| Hoarding | Hoarding Items 1 (“I found it difficult to throw things away.”) and 2 (“I collected things I did not need.”) removed from calculation of scale score as absent from psychiatric/community sample. |
| Pre-mature orgasm | Pre-mature orgasm Items 1 (“During sex, I usually climaxed very quickly”) and 2 (“During sex, I consciously tried to delay orgasm so I could last longer”) absent in psychiatric/community sample, leaving only two items from this scale administered in both samples. Therefore, this scale was excluded from combined analyses. |
| Delusions | Delusions Item 1 (“I could control things with my mind”) removed from calculation of scale score as absent in psychiatric/community sample. |
| Hallucinations | Entire scale excluded from combined analyses as not administered in psychiatric/community sample. |
| Romantic disinterest | Romantic disinterest Item 5 (“I was happiest when I was in a romantic relationship”) removed from calculation of scale score as absent in psychiatric/community sample. |
| Submissiveness | Submissiveness Items 1 (“I allowed people to take advantage of me”) and 2 (“I had trouble standing up for myself”) absent in psychiatric/community sample. In addition, Item 4 worded slightly differently between samples. This left only one item from the Submissiveness scale administered in both samples. Therefore, this scale was excluded from combined analyses. |

**Supplemental Table S2** *Homogeneity and Reliability of HiTOP-SR Scales in Cancer Survivors*

| **HiTOP-SR scale** | **uSRMR [90% CI]** | **Largest absolute residual correlation [90% CI]** | **Omega** |
| --- | --- | --- | --- |
| Agoraphobia | .022 [.017, .027] | .06 [.043, .08] | .91 |
| Antisocial behaviour | .022 [.010, .034] | .07 [.034, .11] | .97 |
| Appetite loss | NA | NA | .89 |
| Binge eating | NA | NA | .91 |
| Bodily distress | .027 [.020, .034] | .06 [.043, .08] | .90 |
| Body dissatisfaction | .028 [.023, .033] | .05 [.032, .07] | .92 |
| Body focus | .044 [.039, .049] | .09 [.081, .11] | .94 |
| Callousness | .064 [.054, .074] | .15 [.127, .18] | .95 |
| Checking | .028 [.025, .031] | .06 [.054, .07] | .94 |
| Cleaning | .021 [.015, .028] | .06 [.04, .08] | .94 |
| Cognitive problems | .013 [.010, .016] | .03 [.02, .03] | .96 |
| Conversion symptoms | .025 [.017, .033] | .07 [.04, .11] | .91 |
| Counting | .065 [.055, .075] | .12 [.10, .14] | .93 |
| Dietary restraint | .052 [.045, .059] | .12 [.11, .14] | .91 |
| Difficulty reaching orgasm | NA | NA | .91 |
| Disease conviction | .019 [.014, .024] | .05 [.04, .07] | .92 |
| Deceitfulness | .016 [.013, .019] | .03 [.02, .04] | .90 |
| Manipulativeness | .014 [.006, .022] | .03 [.01, .04] | .91 |
| Disorganisation | .056 [.049, .063] | .14 [.11, .16] | .90 |
| Dissociation | .022 [.015, .029] | .05 [.03, .07] | .92 |
| Anhedonia | NA | NA | .95 |
| Anxious worry | NA | NA | .93 |
| Depressed mood | .011 [.008, .014] | .02 [.02, .03] | .95 |
| Lassitude | NA | NA | .92 |
| Shame/guilt | NA | NA | .93 |
| Domineering | .013 [.010, .016] | .03 [.02, .05] | .94 |
| Eccentricity | .032 [.025, .039] | .10 [.07, .12] | .93 |
| Affective lability | NA | NA | .92 |
| Angry hostility | .003 [-.002, .008] | .01 [.003, .02] | .92 |
| Irritability | .003 [.001, .005] | .01 [.002, .01] | .95 |
| Entitlement | .054 [.046, .062] | .16 [.13, .20] | .85 |
| Excessive exercise | .035 [.027, .043] | .11 [.08, .13] | .89 |
| Excoriation | NA | NA | .93 |
| Exhibitionism | .023 [.018, .028] | .05 [.04, .06] | .94 |
| Fantasy proneness | .038 [.031, .045] | .10 [.08, .13] | .93 |
| Food selectivity | .005 [.000, .010] | .01 [.002, .02] | .91 |
| Gambling | .016 [.011, .021] | .05 [.03, .06] | .97 |
| Gaming | .004 [-.001, .009] | .01 [.002, .02] | .94 |
| Grandiosity | .042 [.037, .047] | .11 [.09, .13] | .91 |
| Health anxiety | .010 [.007, .013] | .02 [.02, .03] | .94 |
| Hoarding | .067 [.055, .079] | .17 [.14, .21] | .92 |
| Hyperdeliberation | .034 [.031, .037] | .08 [.07, .08] | .95 |
| Hypervigilance | .046 [.039, .053] | .10 [.08, .11] | .94 |
| Insomnia | .016 [.011, .021] | .03 [.02, .04] | .91 |
| Low sexual arousal | NA | NA | .94 |
| Low sexual interest | NA | NA | .96 |
| Manic energy | .028 [.021, .035] | .08 [.07, .10] | .96 |
| Cynicism | .023 [.020, .026] | .05 [.04, .06] | .92 |
| Suspiciousness | .000 [.000, .000] | .002 [-.02, .02] | .90 |
| Muscle building | .031 [.023, .039] | .08 [.05, .11] | .92 |
| Nightmares | NA | NA | .91 |
| Non-persistence | .018 [.015, .021] | .04 [.03, .04] | .93 |
| Non-planfulness | .027 [.022, .032] | .04 [.04, .05] | .93 |
| NSSI | .015 [.010, .020] | .03 [.02, .05] | .94 |
| Oppositionality | .032 [.025, .039] | .07 [.05, .09] | .95 |
| Panic | .053 [.045, .061] | .11 [.09, .12] | .92 |
| Paraphilias | .010 [-.003, .023] | .03 [.002, .05] | .92 |
| Perfectionism | .019 [.014, .024] | .04 [.02, .05] | .95 |
| Premature orgasm | .009 [.001, .017] | .02 [.003, .04] | .87 |
| Problematic shopping | .030 [.022, .038] | .08 [.05, .10] | .90 |
| Purging | NA | NA | .89 |
| Delusions | .020 [.012, .028] | .05 [.03, .06] | .93 |
| Hallucinations | .016 [.012, .028] | .04 [.004, .07] | .92 |
| Restlessness | .014 [.011, .017] | .04 [.03, .05] | .95 |
| Restricted affectivity | .048 [.041, .055] | .12 [.10, .15] | .94 |
| Restricted eating | .016 [.009, .023] | .03 [.02, .05] | .91 |
| Rigidity | .024 [.017, .031] | .08 [.06, .10] | .90 |
| Risk aversion | .029 [.026, .032] | .06 [.05, .07] | .95 |
| Risk taking | .010 [.007, .013] | .03 [.02, .03] | .93 |
| Risky sex | .012 [.002, .022] | .04 [.01, .06] | .84 |
| Romantic disinterest | .041 [.033, .049] | .09 [.07, .11] | .90 |
| Sex related substance use | .015 [.010, .020] | .03 [.02, .05] | .92 |
| Sexual distress | .008 [.005, .011] | .02 [.01, .02] | .95 |
| Sexual pain | NA | NA | .94 |
| Social aggression | .047 [.031, .063] | .11 [.06, .17] | .90 |
| Social aloofness | .008 [.005, .011] | .02 [.01, .03] | .94 |
| Social anxiety | .036 [.031, .041] | .10 [.08, .11] | .94 |
| Somatic preoccupation | .021 [.019, .023] | .05 [.05, .06] | .97 |
| Animal phobia | .031 [.026, .036] | .10 [.08, .11] | .88 |
| Blood/injection phobia | NA | NA | .86 |
| Situational phobia | .041 [.031, .051] | .06 [.04, .08] | .82 |
| Submissiveness | .017 [.012, .022] | .04 [.03, .05] | .94 |
| Suicidality | .031 [.002, .060] | .08 [.00, .17] | .79 |
| Trauma reactions | .061 [.053, .069] | .12 [.10, .13] | .97 |
| Trichotillomania | NA | NA | .92 |
| Well-being | .003 [.000, .006] | .01 [.00, .02] | .93 |
| Workaholism | .017 [.014, .020] | .04 [.03, .05] | .93 |

**Supplemental Table S3** *Loading Matrix for 11-Factor Solution*

| **HiTOP-SR scale** | *Exec & Beh Dys* | *Mal Imp Exp* | *Emo Dys* | *Som Prob* | *Low App & Int* | *Low Sex Func* | *Detach* | *Phobias* | *Disaffil Antag* | *Eat Path* | *Anan* |
| --- | --- | --- | --- | --- | --- | --- | --- | --- | --- | --- | --- |
| Non-persistence | 0.64 | -0.04 | 0.02 | 0.05 | 0.09 | 0.07 | 0.10 | -0.06 | 0.13 | -0.08 | 0.12 |
| Disorganisation | 0.61 | 0.07 | 0.02 | 0.06 | 0.15 | 0.05 | 0.04 | 0.05 | 0.02 | -0.06 | 0.00 |
| Hoarding | 0.58 | 0.03 | -0.04 | -0.03 | -0.01 | 0.06 | -0.04 | 0.18 | 0.07 | 0.15 | -0.01 |
| Problematic shopping | 0.49 | 0.01 | 0.06 | -0.06 | -0.05 | 0.04 | -0.07 | 0.29 | -0.01 | 0.21 | -0.08 |
| Non-planfulness | 0.42 | 0.06 | 0.17 | 0.14 | 0.08 | 0.01 | -0.11 | 0.05 | 0.26 | 0.01 | -0.20 |
| Cognitive problems | 0.38 | -0.04 | 0.22 | 0.26 | 0.22 | 0.07 | 0.07 | 0.00 | -0.06 | 0.01 | 0.03 |
| Binge eating | 0.34 | 0.10 | 0.03 | 0.13 | -0.34 | 0.08 | 0.17 | 0.03 | 0.07 | 0.31 | -0.03 |
| Lassitude | 0.34 | -0.08 | 0.18 | 0.28 | 0.09 | 0.12 | 0.23 | -0.10 | -0.05 | 0.00 | 0.18 |
| Deceitfulness | 0.33 | 0.20 | 0.15 | 0.02 | -0.02 | 0.03 | -0.06 | -0.02 | 0.24 | 0.01 | -0.05 |
| Fantasy proneness | 0.32 | 0.18 | 0.07 | 0.25 | 0.09 | 0.02 | -0.01 | -0.05 | 0.07 | 0.09 | 0.17 |
| Checking | 0.31 | -0.01 | 0.11 | 0.19 | 0.17 | 0.06 | -0.02 | 0.15 | -0.07 | 0.07 | 0.18 |
| Excoriation | 0.30 | 0.28 | -0.09 | -0.07 | 0.04 | 0.03 | 0.05 | 0.11 | -0.10 | 0.06 | 0.17 |
| Restlessness | 0.28 | 0.09 | 0.02 | 0.02 | 0.22 | 0.01 | -0.03 | 0.14 | 0.06 | 0.10 | 0.04 |
| Paraphilias | -0.07 | 0.55 | 0.03 | 0.07 | -0.07 | 0.05 | -0.07 | 0.05 | 0.11 | 0.07 | -0.01 |
| Antisocial behaviour | -0.03 | 0.49 | -0.06 | 0.00 | 0.03 | -0.02 | 0.07 | 0.18 | 0.12 | 0.08 | -0.08 |
| Risky sex | -0.01 | 0.46 | 0.16 | 0.01 | 0.15 | 0.02 | -0.09 | -0.08 | 0.00 | -0.02 | -0.03 |
| Sex related substance use | 0.04 | 0.43 | 0.07 | -0.02 | 0.09 | 0.19 | -0.08 | 0.00 | 0.00 | -0.03 | -0.05 |
| NSSI | 0.19 | 0.41 | 0.03 | 0.00 | 0.18 | 0.02 | 0.14 | 0.00 | -0.07 | 0.05 | 0.02 |
| Gambling | 0.02 | 0.41 | -0.16 | 0.05 | -0.03 | 0.10 | 0.04 | 0.22 | 0.10 | -0.01 | -0.06 |
| Delusions | -0.10 | 0.37 | -0.03 | -0.03 | 0.15 | 0.00 | -0.08 | 0.20 | 0.12 | 0.07 | 0.07 |
| Gaming | 0.11 | 0.34 | -0.13 | 0.01 | 0.02 | -0.04 | 0.04 | 0.05 | 0.17 | 0.02 | 0.07 |
| Dissociation | 0.18 | 0.31 | 0.11 | 0.20 | 0.20 | 0.02 | 0.08 | 0.06 | 0.03 | 0.01 | 0.05 |
| Trichotillomania | 0.22 | 0.31 | -0.03 | -0.12 | 0.07 | 0.04 | 0.06 | 0.13 | 0.00 | 0.00 | 0.11 |
| Risk taking | 0.09 | 0.30 | 0.04 | -0.05 | 0.25 | 0.00 | -0.27 | -0.11 | 0.15 | 0.15 | -0.18 |
| Manipulativeness | 0.13 | 0.29 | 0.08 | 0.02 | 0.00 | -0.01 | -0.10 | 0.00 | 0.28 | 0.02 | -0.07 |
| Eccentricity | 0.24 | 0.29 | 0.03 | 0.21 | 0.15 | 0.00 | -0.08 | -0.04 | 0.25 | -0.03 | 0.07 |
| Irritability | 0.09 | -0.09 | 0.58 | 0.07 | 0.02 | 0.07 | 0.15 | 0.11 | 0.14 | 0.06 | 0.01 |
| Anger hostility | -0.04 | 0.03 | 0.55 | 0.11 | 0.01 | 0.06 | 0.01 | 0.19 | 0.22 | 0.04 | -0.07 |
| Affective lability | 0.03 | 0.13 | 0.47 | 0.14 | 0.17 | 0.04 | 0.01 | 0.11 | 0.07 | 0.06 | -0.02 |
| Anxious worry | 0.16 | -0.01 | 0.44 | 0.12 | 0.20 | 0.02 | 0.16 | 0.07 | -0.16 | 0.01 | 0.21 |
| Suspiciousness | 0.00 | 0.12 | 0.32 | 0.06 | 0.09 | 0.04 | -0.01 | 0.27 | 0.23 | 0.05 | 0.04 |
| Entitlement | 0.23 | 0.12 | 0.32 | -0.06 | -0.07 | 0.07 | -0.15 | -0.03 | 0.30 | 0.08 | 0.12 |
| Bodily distress | 0.09 | -0.03 | 0.06 | 0.55 | 0.12 | 0.12 | 0.07 | 0.14 | 0.00 | 0.08 | -0.01 |
| Health anxiety | 0.01 | 0.05 | 0.07 | 0.53 | -0.02 | 0.09 | -0.03 | 0.14 | 0.02 | 0.05 | 0.15 |
| Somatic preoccupation | -0.05 | -0.09 | -0.04 | 0.49 | 0.01 | 0.09 | -0.20 | 0.06 | 0.04 | 0.19 | 0.22 |
| Suicidality | 0.03 | 0.19 | 0.12 | 0.40 | 0.09 | 0.08 | 0.14 | -0.03 | 0.08 | -0.01 | 0.05 |
| Panic | 0.10 | 0.09 | 0.12 | 0.37 | 0.24 | 0.05 | 0.03 | 0.22 | -0.05 | -0.03 | 0.05 |
| Disease conviction | 0.02 | 0.12 | 0.07 | 0.37 | 0.08 | 0.04 | 0.03 | 0.20 | 0.09 | 0.04 | 0.03 |
| Conversion symptoms | -0.09 | 0.31 | -0.10 | 0.34 | 0.15 | 0.10 | -0.01 | 0.20 | 0.06 | 0.04 | -0.07 |
| Workaholism | 0.00 | 0.05 | 0.07 | -0.27 | 0.26 | 0.05 | -0.14 | 0.05 | 0.03 | 0.22 | 0.15 |
| Insomnia | 0.14 | -0.08 | 0.09 | 0.27 | 0.13 | 0.14 | 0.16 | 0.06 | 0.03 | 0.09 | -0.06 |
| Restricted eating | 0.07 | 0.04 | -0.01 | -0.06 | 0.67 | 0.05 | 0.02 | 0.03 | 0.03 | 0.11 | -0.01 |
| Appetite loss | -0.03 | -0.03 | 0.01 | 0.17 | 0.66 | 0.08 | 0.02 | 0.08 | -0.02 | -0.03 | -0.08 |
| Manic energy | 0.10 | 0.23 | 0.08 | -0.03 | 0.28 | -0.08 | -0.22 | 0.12 | 0.13 | 0.09 | -0.01 |
| Nightmares | 0.13 | 0.14 | 0.08 | 0.18 | 0.27 | 0.06 | -0.01 | 0.12 | -0.05 | -0.01 | 0.10 |
| Low sexual arousal | -0.04 | -0.06 | -0.03 | 0.00 | 0.00 | 0.96 | -0.04 | 0.00 | 0.03 | -0.03 | -0.04 |
| Difficulty reaching orgasm | 0.03 | 0.10 | 0.03 | -0.01 | 0.03 | 0.75 | -0.08 | -0.06 | -0.07 | -0.03 | -0.02 |
| Low sexual interest | 0.04 | -0.20 | -0.12 | -0.02 | 0.00 | 0.67 | 0.14 | 0.07 | 0.15 | 0.03 | 0.05 |
| Sexual distress | 0.02 | 0.23 | 0.14 | -0.02 | -0.06 | 0.63 | 0.04 | -0.08 | -0.11 | 0.03 | 0.09 |
| Sexual pain | -0.03 | 0.18 | -0.02 | 0.08 | -0.02 | 0.52 | -0.02 | 0.16 | -0.13 | 0.02 | 0.01 |
| Grandiosity | -0.03 | 0.07 | -0.02 | 0.01 | 0.04 | -0.05 | -0.74 | -0.02 | 0.01 | 0.11 | 0.14 |
| Low well-being | 0.04 | 0.03 | 0.18 | 0.03 | 0.01 | 0.03 | 0.69 | -0.01 | 0.07 | -0.05 | -0.06 |
| Exhibitionism | 0.22 | 0.20 | 0.19 | 0.00 | 0.02 | 0.01 | -0.44 | -0.07 | 0.08 | 0.13 | -0.04 |
| Domineering | 0.05 | 0.06 | 0.27 | -0.16 | 0.04 | 0.07 | -0.44 | 0.02 | 0.21 | 0.11 | 0.10 |
| Depressed mood | 0.05 | 0.12 | 0.39 | 0.19 | 0.14 | 0.05 | 0.43 | -0.11 | 0.02 | 0.07 | 0.15 |
| Anhedonia | 0.07 | 0.04 | 0.12 | 0.20 | 0.14 | 0.15 | 0.41 | -0.08 | 0.23 | 0.02 | 0.14 |
| Social aloofness | 0.05 | -0.13 | -0.06 | 0.11 | 0.12 | 0.10 | 0.37 | 0.08 | 0.35 | 0.01 | 0.21 |
| Shame/guilt | 0.24 | 0.11 | 0.35 | 0.01 | 0.09 | 0.04 | 0.36 | -0.07 | 0.01 | 0.17 | 0.12 |
| Social anxiety | 0.20 | 0.02 | 0.03 | -0.02 | 0.11 | 0.07 | 0.32 | 0.20 | 0.09 | 0.00 | 0.24 |
| Situational phobia | 0.00 | 0.02 | 0.11 | 0.06 | 0.04 | 0.01 | -0.01 | 0.59 | -0.02 | -0.04 | 0.10 |
| Animal phobia | 0.02 | 0.00 | 0.05 | 0.03 | 0.01 | 0.05 | -0.03 | 0.57 | -0.03 | 0.00 | 0.04 |
| Blood/injection phobia | 0.03 | 0.11 | -0.03 | 0.05 | 0.05 | 0.05 | -0.06 | 0.44 | -0.05 | -0.01 | 0.07 |
| Agoraphobia | 0.01 | 0.05 | 0.03 | 0.25 | 0.16 | 0.08 | 0.07 | 0.43 | 0.00 | -0.02 | 0.00 |
| Food selectivity | 0.13 | 0.04 | -0.06 | 0.03 | 0.27 | 0.00 | 0.01 | 0.30 | 0.04 | 0.06 | 0.09 |
| Counting | 0.09 | 0.24 | -0.04 | 0.11 | 0.10 | 0.01 | -0.06 | 0.29 | 0.02 | 0.08 | 0.08 |
| Callousness | -0.02 | 0.09 | 0.08 | 0.08 | -0.02 | 0.09 | 0.12 | -0.02 | 0.56 | 0.04 | -0.03 |
| Social aggression | 0.14 | 0.17 | 0.21 | -0.02 | -0.07 | 0.03 | -0.11 | 0.05 | 0.43 | -0.07 | -0.05 |
| Oppositionality | 0.31 | 0.15 | 0.03 | 0.02 | 0.05 | -0.02 | -0.12 | -0.07 | 0.42 | -0.02 | -0.03 |
| Romantic disinterest | 0.00 | -0.05 | -0.10 | -0.07 | 0.12 | 0.08 | 0.31 | 0.06 | 0.40 | 0.13 | 0.08 |
| Restricted affectivity | 0.05 | 0.05 | -0.07 | 0.08 | 0.20 | 0.14 | 0.20 | -0.05 | 0.37 | 0.15 | 0.10 |
| Rigidity | 0.19 | 0.07 | 0.22 | -0.13 | 0.03 | 0.01 | -0.08 | 0.07 | 0.36 | -0.04 | 0.25 |
| Cynicism | -0.05 | 0.06 | 0.28 | 0.04 | 0.04 | 0.04 | -0.03 | 0.20 | 0.35 | 0.03 | 0.20 |
| Muscle building | -0.05 | 0.10 | -0.01 | -0.02 | 0.05 | -0.10 | -0.16 | -0.10 | 0.09 | 0.58 | 0.08 |
| Dietary restraint | -0.21 | -0.11 | -0.12 | 0.21 | 0.08 | 0.08 | -0.13 | 0.00 | 0.01 | 0.55 | 0.07 |
| Body dissatisfaction | 0.25 | -0.09 | 0.22 | 0.04 | -0.12 | 0.06 | 0.25 | 0.10 | -0.08 | 0.50 | -0.03 |
| Body focus | 0.08 | 0.02 | 0.24 | 0.03 | 0.12 | 0.06 | -0.25 | 0.07 | -0.12 | 0.42 | 0.01 |
| Excessive exercise | -0.08 | 0.23 | -0.13 | 0.00 | 0.19 | 0.00 | -0.04 | -0.01 | 0.08 | 0.40 | 0.02 |
| Purging | 0.00 | 0.29 | -0.09 | -0.07 | 0.07 | 0.07 | 0.11 | 0.17 | -0.03 | 0.30 | 0.00 |
| Hyperdeliberation | -0.07 | -0.02 | -0.06 | 0.04 | 0.00 | 0.05 | -0.22 | -0.06 | 0.00 | 0.08 | 0.65 |
| Risk aversion | -0.01 | -0.06 | -0.03 | 0.10 | -0.14 | 0.00 | 0.03 | 0.24 | 0.02 | -0.01 | 0.63 |
| Perfectionism | 0.10 | 0.08 | 0.16 | -0.27 | 0.22 | 0.02 | -0.11 | -0.01 | 0.00 | 0.22 | 0.35 |
| Hypervigilance | 0.15 | 0.07 | 0.26 | 0.11 | 0.10 | -0.01 | -0.01 | 0.22 | 0.06 | -0.01 | 0.30 |
| Trauma reactions | 0.08 | 0.14 | 0.17 | 0.11 | 0.19 | 0.01 | 0.07 | 0.19 | 0.05 | 0.05 | 0.23 |

*Note*. *Exec & Beh Dys* = *Executive & Behavioural Dyscontrol*; *Mal Imp Exp* = *Maladaptive Impulse Expression*; *Emo Dys* = *Emotional Dysregulation*; *Som Prob* = *Somatoform Problems*; *Low App & Int* = *Low Appetite & Intake*; *Low Sex Func* = *Low Sexual Function*; *Detach* = *Detachment*; *Phobias* = *Phobias*; *Disaffil Antag* = *Disaffiliative Antagonism*; *Eat Path* = *Eating Pathology*; *Anan* = *Anankastia*.

**Supplemental Table S4** *Inter-Factor Correlations for 11-Factor Solution*

| **Psychopathology dimensions** | *Exec & Beh Dys* | *Mal Imp Exp* | *Emo Dys* | *Som Prob* | *Low App & Int* | *Low Sex Func* | *Detach* | *Phobias* | *Disaffil Antag* | *Eat Path* | *Anan* |
| --- | --- | --- | --- | --- | --- | --- | --- | --- | --- | --- | --- |
| *Exec & Beh Dys* | 1.00 | - | - | - | - | - | - | - | - | - | - |
| *Mal Imp Exp* | .33 | 1.00 | - | - | - | - | - | - | - | - | - |
| *Emo Dys* | .53 | .28 | 1.00 | - | - | - | - | - | - | - | - |
| *Som Prob* | .24 | .13 | .30 | 1.00 | - | - | - | - | - | - | - |
| *Low App & Int* | .32 | .38 | .31 | .37 | 1.00 | - | - | - | - | - | - |
| *Low Sex Func* | .26 | .15 | .21 | .38 | .27 | 1.00 | - | - | - | - | - |
| *Detach* | .22 | -.18 | .17 | .28 | .06 | .21 | 1.00 | - | - | - | - |
| *Phobias* | .25 | .22 | .20 | .36 | .27 | .27 | .07 | 1.00 | - | - | - |
| *Disaffil Antag* | .32 | .32 | .28 | .15 | .23 | .22 | .04 | .18 | 1.00 | - | - |
| *Eat Path* | .26 | .25 | .23 | .16 | .24 | .21 | -.14 | .22 | .19 | 1.00 | - |
| *Anan* | .21 | .04 | .25 | .26 | .27 | .22 | .05 | .25 | .16 | .27 | 1.00 |

*Note*. *Exec & Beh Dys* = *Executive & Behavioural Dyscontrol*; *Mal Imp Exp* = *Maladaptive Impulse Expression*; *Emo Dys* = *Emotional Dysregulation*; *Som Prob* = *Somatoform Problems*; *Low App & Int* = *Low Appetite & Intake*; *Low Sex Func* = *Low Sexual Function*; *Detach* = *Detachment*; *Phobias* = *Phobias*; *Disaffil Antag* = *Disaffiliative Antagonism*; *Eat Path* = *Eating Pathology*; *Anan* = *Anankastia*.

**Supplemental Table S5** *Configural Invariance of Psychopathology Dimensions Between Cancer-Survivor and Community/Psychiatric Samples*

|  | **Cancer survivors** | | | **Non-cancer-specific sample** | | |
| --- | --- | --- | --- | --- | --- | --- |
| **Psychopathology dimensions** | **uSRMR [90% CI]** | **Largest absolute residual correlation [90% CI]** | **Reliability** | **uSRMR [90% CI]** | **Largest absolute residual correlation [90% CI]** | **Reliability** |
| *Executive & Behavioural Dyscontrol* | .060 [.053, .067] | Problematic shopping and hoarding: .25 [.19, .32] | ω = .89 | .072 [.062, .082] | Problematic shopping and hoarding: .41 [.34, .47] | ω = .90 |
| *Maladaptive Impulse Expression* | .038 [.023, .053] | .13 [.07, .20] | ω = .90 | .053 [.032, .074] | .24 [.05, .44] | ω = .78 |
| *Emotional Dysregulation* | .065 [.055, .075] | Guilt/shame and depressed mood: .21 [.15, .27] | ω = .91 | .055 [.047, .063] | Guilt/shame and depressed mood: .14 [.09, .18] | ω = .92 |
| *Somatoform Problems* | .037 [.027, .047] | .12 [.08, .16] | ω = .85 | .045 [.032, .058] | .12 [.04, .20] | ω = .88 |
| *Low Appetite & Intake* (EFA) | - | - | α =.44 | - | - | α = .44 |
| Restricted eating | λ = .61 | |  | λ = 1.00 | |  |
| Appetite loss | λ = 1.00 | |  | λ = .76 | |  |
| Binge eating | λ = -.06 | |  | λ = .06 | |  |
| *Low Sexual Function* | .062 [.047, .077] | Sexual pain and sexual distress: .21 [.15, .26] | ω = .84 | .034 [.021, .047] | Low sexual interest and difficulty reaching orgasm: .09 [.06, .12] | ω = .83 |
| *Detachment* | .147 [.121, .173] | Domineering and exhibitionism: .58 [.46, .69] | ω = .72 | .113 [.098, .128] | Domineering and exhibitionism: .41 [.34, .49] | ω = .84 |
| *Phobias* | .015 [.000, .030] | .05 [.01, .10] | ω = .79 | .026 [.013, .039] | .06 [.01, .10] | ω = .77 |
| *Disaffiliative Antagonism* | .064 [.052, .076] | Social aloofness and romantic disinterest: .24 [.18, .30] | ω = .76 | .050 [.040, .060] | Social aloofness and romantic disinterest: .16 [.10, .21] | ω = .82 |
| *Eating Pathology* | .101 [.088, .114] | Binge eating and body dissatisfaction: .36 [.30, .43] | ω = .66 | .081 [.068, .094] | Binge eating and body dissatisfaction: .30 [.24, .37] | ω = .68 |
| *Anankastia* | .045 [.030, .060] | .09 [.07, .11] | ω = .69 | .061 [.046, .076] | .11 [.06, .18] | ω = .64 |

*Note*. Green cells denote metrics that satisfied our *a priori* criteria for configural invariance, whereas red cells indicate metrics suggestive of possible configural non-invariance. For *Low Appetite & Intake*, a one-factor CFA model failed to converge. As an alternative, one-factor exploratory factor analyses were fit to the three indicators in each sample; the resulting factor loadings were inspected qualitatively, and coefficient alpha (an alternative reliability coefficient) was computed for the summed total score. uSRMR = unbiased standardised root mean squared residual.

**Supplemental Table S6** *Metric Invariance of Psychopathology Dimensions Between Cancer-Survivor and Community/Psychiatric Samples*

| **Psychopathology dimension** | **Δχ^2^** | ***p*** | **ΔCFI** | **ΔRMSEA** | **Potential non-invariant indicators (based on Δλ ≥ .10)** | | |
| --- | --- | --- | --- | --- | --- | --- | --- |
|  |  |  |  |  | **HiTOP-SR scale** | **λ_Cancer_** | **λ_Non-cancer_** |
| *Executive & Behavioural Dyscontrol* | 16.68 | .118 | -.001 | -.006 | Lack of evidence for metric non-invariance | | |
| *Maladaptive Impulse Expression* | 38.00 | <.001 | -.030 | .007 | Paraphilias | .84 | .57 |
|  |  |  |  |  | Risky sex | .61 | .47 |
|  |  |  |  |  | Sex related substance use | .70 | .50 |
|  |  |  |  |  | NSSI | .74 | .60 |
|  |  |  |  |  | Gambling | .62 | .41 |
|  |  |  |  |  | Reality distortion | .78 | .67 |
|  |  |  |  |  | Gaming | .58 | .40 |
|  |  |  |  |  | Trichotillomania | .65 | .28 |
| *Emotional Dysregulation* | 34.89 | <.001 | -.006 | -.012 | Entitlement | .61 | .46 |
| *Somatic Problems* | 50.95 | <.001 | -.021 | .008 | Bodily distress | .75 | .86 |
| *Low Appetite & Intake* (EFA) | - | - | - | - | Restricted eating | .61 | .100 |
|  |  |  |  |  | Appetite loss | 1.00 | .76 |
| *Low Sexual Function* | 6.73 | .151 | -.002 | -.022 | No scales differed in their loadings by .10 or more | | |
| *Detachment* | 40.65 | <.001 | -.008 | -.010 | Grandiosity | -.19 | -.55 |
|  |  |  |  |  | Low well-being | .51 | .72 |
|  |  |  |  |  | Exhibitionism | -.18 | -.01 |
| *Phobias* | 27.14 | <.001 | -.025 | .020 | Blood-injection phobia | .68 | .48 |
| *Disaffiliative Antagonism* | 14.38 | .045 | -.005 | -.006 | Callousness | .62 | .78 |
|  |  |  |  |  | Social aloofness | .47 | .62 |
| *Eating Pathology* | 23.91 | <.001 | -.026 | -.009 | Dietary restraint | .51 | .69 |
|  |  |  |  |  | Excessive exercise | .66 | .53 |
|  |  |  |  |  | Purging | .59 | .39 |
| *Anankastia* | 5.08 | .166 | -.004 | -.044 | Risk aversion | .62 | .76 |
|  |  |  |  |  | Perfectionism | .55 | .40 |
|  |  |  |  |  | Hypervigilance | .60 | .49 |

*Note*. Green cells denote metrics that satisfied our *a priori* criteria for metric invariance, whereas red cells indicate metrics suggestive of possible metric non-invariance. For *Low Appetite & Intake*, a one-factor CFA model failed to converge. As an alternative, one-factor exploratory factor analyses were fit to the three indicators in each sample; the resulting factor loadings were inspected qualitatively. CFI = Comparative Fit Index; RMSEA = Root Mean Square Error of Approximation; HiTOP-SR = Hierarchical Taxonomy of Psychopathology – Self-Report; EFA = exploratory factor analysis.

**Supplemental Figure S1** *HiTOP-SR Overall Cancer Sample (n=728) and Normative Data Comparisons (n=780)*

**
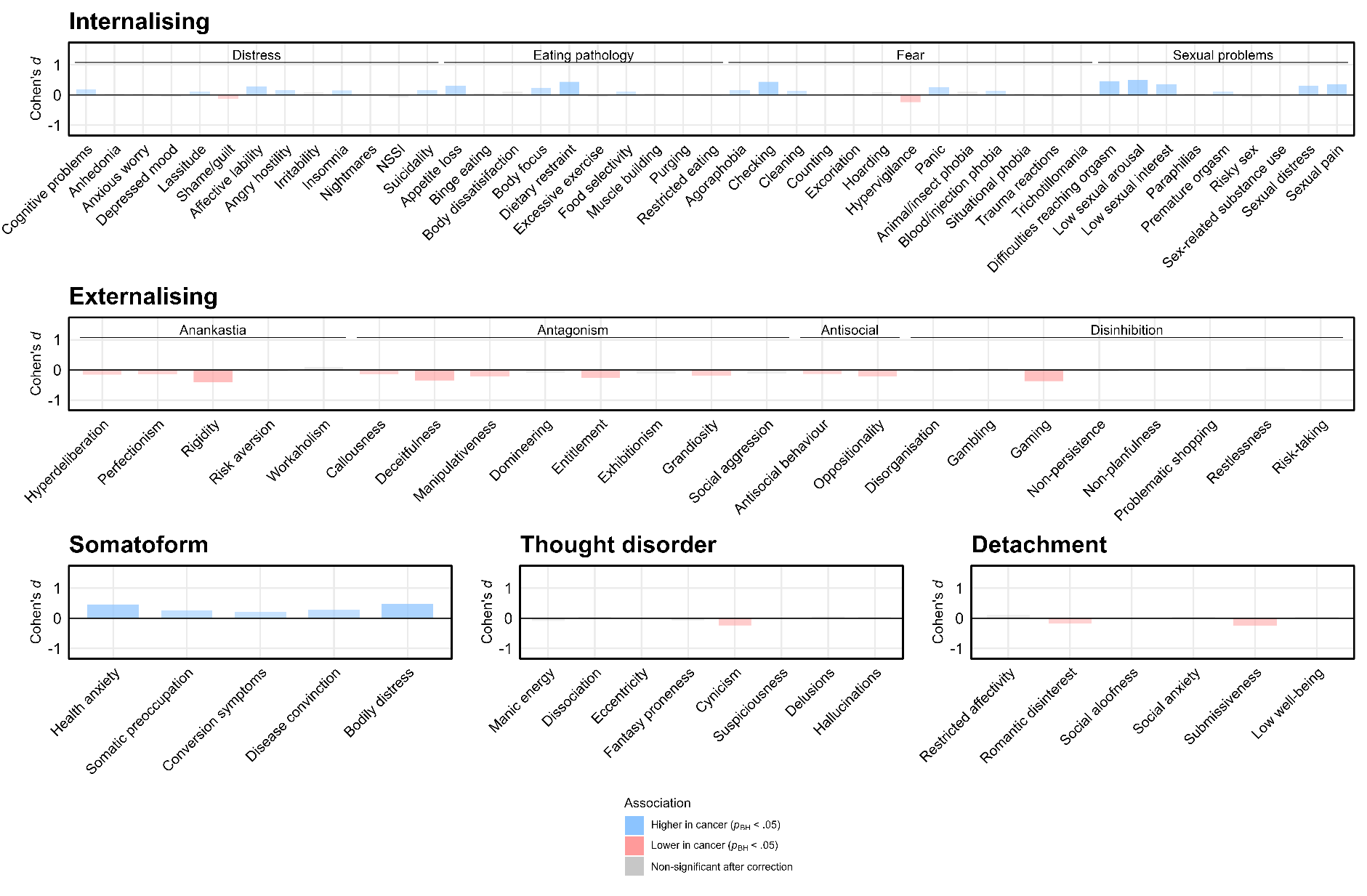
**

*Note*. HiTOP-SR scales are presented according to the spectra to which they have been rationally assigned by the HiTOP Consortium’s Measure Development Workgroup, given that the empirical structure of the HiTOP-SR remains an area of active investigation. Subheadings within the Internalising and Externalising spectra reflect subfactors from the established HiTOP model.
